# Health state utility values ranges across varying stages and severity of type 2 diabetes-related complications: A systematic review

**DOI:** 10.1371/journal.pone.0297589

**Published:** 2024-04-04

**Authors:** Michelle Hwee Pheng Tan, Siew Chin Ong, Nurul Ain Mohd Tahir, Adliah Mhd Ali, Norlaila Mustafa

**Affiliations:** 1 Pharmacy Department, Hospital Canselor Tuanku Muhriz, Universiti Kebangsaan Malaysia, Kuala Lumpur, Malaysia; 2 Discipline of Social and Administrative Pharmacy, School of Pharmaceutical Science, Universiti Sains Malaysia, Pulau Pinang, Malaysia; 3 Department of Pharmacy, Kampus Kuala Lumpur Universiti Kebangsaan Malaysia, Kuala Lumpur, Malaysia; 4 Department of Medicine, Universiti Kebangsaan Malaysia, Kuala Lumpur, Malaysia; Centre Hospitalier Sud Francilien, FRANCE

## Abstract

**Introduction:**

Health state utility values (HSUV) for Type 2 diabetes mellitus (T2DM) complications are useful in economic evaluations to determine cost effectiveness of an intervention. However, there is a lack of reference ranges for different severity and stages of individual complications. This study aimed to provide an overview of HSUV decrement ranges for common T2DM complications focusing on different severity and stages of complications.

**Method:**

A systematic search was conducted in MEDLINE, SCOPUS, WEB OF SCIENCE. (Jan 2000 to April 2022). Included studies for HSUV estimates were from outpatient setting, regardless of treatment types, complication stages, regions and HRQoL instruments. Health Related Quality of Life (HRQoL) outcomes was to be presented as HSUV decrement values, adjusted according to social demographics and comorbidities. Adjusted HSUV decrements were extracted and compiled according to individual complications. After which, subsequently grouped into mild or severe category for comparison.

**Results:**

Searches identified 35 studies. The size of the study population ranged from 160 to 14,826. The HSUV decrement range was widest for cerebrovascular disease (stroke): -0.0060 to -0.0780 for mild stroke and -0.035 to -0.266 for severe stroke; retinopathy: mild (-0.005 to -0.0862), moderate (-0.0030 to -0.1845) and severe retinopathy (-0.023 to -0.2434); amputation: (-0.1050 to -0.2880). Different nature of complication severity defined in studies could be categorized into: those with acute nature, chronic with lasting effects, those with symptoms at early stage or those with repetitive frequency or episodes.

**Discussion:**

Overview of HSUV decrement ranges across different stages of each T2DM diabetes-related complications shows that chronic complications with lasting impact such as amputation, severe stroke with sequelae and severe retinopathy with blindness were generally associated with larger HSUV decrement range. Considerable heterogeneities exist across the studies. Promoting standardized complication definitions and identifying the most influential health state stages on HSUV decrements may assist researchers for future cost-effectiveness studies.

## Introduction

The global diabetes prevalence is predicted to rise 19% by 2030, and another 22% by 2045. It is alarming that three out of four adults in low-and middle-income countries are living with diabetes. Between 2000 and 2019, age-standardized mortality rates from diabetes alone increased by 3%, with 13% solely from lower-middle-income countries [1]. Furthermore, diabetes-related complications are the leading cause of global blindness, diabetes-related deaths and end stage renal failure (ESRF). On average, patients with diagnosed diabetes spend approximately 2.3 times higher on medical expenditures than those without diabetes. In the U.S., national health care costs attributable to diabetes have increased by 35% ($80 billion) in the past 10 years—from $227 billion in 2012 to $307 billion in 2022. This highlights the immense burden of diabetes on the society [2].

In this context, economic evaluations have been a dominant feature in the resource allocation of diabetes prevention and management. Quality-adjusted life years (QALYs) are commonly used outcome measures required for inputs into diabetes progression simulation models. The weights (or commonly known as health state utility values, HSUV) used to calculate QALYs are based on preference-based measures of health-related quality of life (HRQOL). Published studies in patients with diabetes have assessed the impact of complications using several different approaches such as direct methods: time-trade off (TTO), standard gamble (SG) and indirect methods: 5-level EQ-5D version, (ED-5D-5L), Short-form 6-dimensional (SF-6D), Self-administered Quality of Well-Being Index (QWB-SA) and Health Utilities Index Mark 3 (HUI-3).

Previous systematic reviews to elicit HSUVs for all major diabetes complications have found considerable heterogeneity and thus were not able to conduct meta-analyses to pool utility values together [3]. In recent years, there was an attempt to pool HSUVs for all major diabetes complications specifically in the East and Southeast Asia regions. However, pooled values presented much heterogeneity due to different populations, valuation instruments and lacked complication stage and severity details [4, 5]. For example, a systematic review conducted to evaluate utility scores for economic modelling in T2DM reported utility score for myocardial infarction was 0.057 by O’Reilly (Canada) [6], 0.055 by Clarke et al (U.K.) [7], 0.041 by Glasziou (Australia) [8, 9], 0.007 by Lee (Korea) [9]. The large range of difference makes selection of HSUV difficult for use in pharmacoeconomic studies as it may have considerable effect on outcome of economic evaluations [10]. Biased HSUVs will result in biased cost-effectiveness analyses and potential misallocation of resources [11].

A motivation for this study is the lack of overview HSUV ranges for individual diabetes complications at their varying severity across all instruments, populations, regions for an overall comparison in one study. A Japanese study had previously demonstrated that utility decrement for diabetic complication varied with its severity and symptoms [12]. This study aims to review the HSUV decrement ranges for individual diabetes complication and how they are defined in individual studies for the outpatient setting.

## Method

This review was conducted according to the Preferred Reporting Items for Systematic Reviews and Meta-Analyses (PRISMA) guidelines and The Professional Society for Health Economics and Outcomes Research (ISPOR) Good practices for outcomes research task force report 2019 [13, 14]. The study protocol was registered with Prospective Register of Systematic Reviews (PROSPERO: CRD42022301337).

### 2.1 Eligibility criteria

Studies included T2DM population in outpatient setting with diabetes-related complications regardless of treatment type, complication stage or severity and region. Studies were to be conducted in adults aged > 18 years old, published in English since January 2000 to April 2022. Both observational and experimental studies which applied direct (standard gamble, TTO) or indirect methods (EQ-5D and all its variants, SF-6D, HUI-3, QWB-SA) to elicitate HSUVs were included.

Non-original studies such as editorials, systematic reviews, meta-analysis, and guidelines were excluded. Quality of life studies associated with intervention or reported without HSUVs were excluded. Studies that reported HSUVs based on hypothetical scenarios or vignettes were excluded. Studies solely reporting HSUV estimates without adjustment for other HRQoL predictors, or direct elicitation from visual analog score (VAS) were excluded.

HRQoL outcomes were to be presented as HSUV decrement (disutility values), preferably adjusted according to social demographics and other predictors of HRQoL. Focusing on HSUV decrements allow broad overview comparison to be made as baseline HSUV will differ for each study depending on valuation methods, tariffs and populations.

### 2.2 Information sources

Computer simulations of economic models allow projection of long-term clinical outcomes, aiding policy makers in deciding on cost-effective interventions for certain populations. Diabetes economic models were identified from Mount Hood Diabetes Challenge Registry to identify complication types with impact on HSUVs [15]. United Kingdom Prospective Diabetes Study Outcomes Model 2 (UKPDS OM2), Cardiff Diabetes Model and Sheffield Diabetes Model were deemed relevant as they characterized macrovascular and microvascular complications including direct consequences of T2DM treatment such as hypoglycemia. Complications identified included cardiovascular or coronary heart disease (including myocardial infarction, (MI) and ischemic heart disease), heart failure, cerebrovascular disease (stroke), amputation, diabetic foot ulcer, nephropathy, end-stage renal failure (ESRF), retinopathy (including blindness), neuropathy and hypoglycemia. Definitions of each complication defined in each study are outlined in Supplementary Material.

The electronic databases Medline, Scopus, Web of Science Core collection (including conference proceedings) were searched. Initial database searches were conducted from December 2021 to February 2022 and subsequently updated to include sources through April 2022.

### 2.3 Search strategy

Our search strategy included keywords such as diabetes, complications (coronary heart disease, heart failure, stroke, nephropathy, retinopathy, neuropathy, amputation, foot ulcer), quality of life, health utility values. ‘Utility value’ was a very generic term which broaden sensitivity of the search. In order to test for completeness, relevant search terms were identified and added to the search strategy by scanning the top retrieved articles for relevant synonyms such as ‘utility scores’ and other similar synonyms. The full search strategy can be found in S1 Table. Included studies were entered into the reference manager Mendeley to remove duplicates. Initial title and abstract screening were conducted by one researcher. Citations were imported into Covidence, a web-based collaboration software platform [16]. A second title and abstract screening with full text screening were independently conducted in Covidence by two researchers. Any decision to exclude were typed in Covidence notepad, allowing discussion among the two reviewers. Unresolved disagreements involved a third reviewer. One reviewer collected data from each study, checked by the second reviewer.

### 2.4 Data extraction (Outcomes)

The data extraction items were created based on Checklist for Reporting Valuation Studies (CREATE), National Institute of Health and Care Excellence (NICE) Technical Support Document, and ISPOR Good Practices for Outcomes Research Task Force Report [11, 14, 17, 18]. Information extracted included study background, patient characteristics, general population norms, data collection and treatment methods, preference weights for determination of HSUV, descriptive statistics for health states valued, synthesis of HSUV in study, regression models and coefficients (S2 File, Table 1).

**Table 1:** Summary of included studies: Characteristics of population and recruitment setting.

Author/Year	Country /Tariff	Mean age/T2DM duration (years)	Comorbidities	N, number of participants	HSUV DM population	HSUV in DM without complica-tion	Self-report (S) or medical record (M)	Respondent recruitment and setting	T2DM Medication (%)	Statistical Methods
Clarke(1), 2002	UK/UK	62.3 / 10.6	NR	3192	0.70	0.79	M	UKPDS study patients. Questionnaires mailed	NR	Tobit model
Coffey (2), 2002	US/NR	57.6 / 10.4	Hypertension, ↑lipid	2041	0.69	NR	S	Outpatient specialty clinics	OHA 39%, insulin 54%	Multiple linear regression
Tabaei (3), 2004	US/NR	56 / 9	Hypertension, ↑lipid	888	NR	NR	S	Outpatient specialty clinics	diet, insulin, OHA	Multiple linear regression
Bagust (4), 2005	UK/UK	67 / 9.9	NR	4641	0.69	0.79	M	Code-2 Study pts: epidemiological registry, Questionnaire-survey	Oral 57.4%; insulin 17.1%	OLS/TTO model
Tung (5), 2005	Taiwan/NR	60–70 / >10	NR	406	0.92	0.94	M	Household registry community survey. Face to face interviews.	OHA and insulin	Multiple linear regression
P. Clarke (6), 2006	UK/UK	61.6 / 6	Hypertension, ↑lipid	4051	0.76	0.85	M	Lipids in Diabetes Study pts. Outpt clinic and GP registers of diabetics	NR	Step wise regression
Maddigan (7), 2006	Canada/Canada	61.7 / 9.4	Cataract, depression	5134	0.90	0.88	M	Canadian Community Health Survey Cycle CCHS (include non-DM). General population survey	insulin users 15.6%	Bootstrap variance
Wexler (8), 2006	U.S/NR	66 / 9.6	Copd, ↑lipid, hypertension	909	0.70	0.84	M	Hospital based clinic, community health centre. General population survey: mail/phone	OHA and/or insulin	General linear modelling-
Smith (9), 2008	US/US	65.6 / 7.1	ESRF, macular edema	2074	0.82	0.94	M	community based population. Random mail survey	OHA and/or insulin	Linear regression
Lloyd (10), 2008	UK/UK	62.2 / NR	Neuropathy, nephropathy	319	0.83	0.77	M	T2DM with DR: general population (advertisement/database). In clinic /survey- face to face interviews/phone	NR	Mixed model and regression analysis
Solli (11), 2010	Norway/UK	64 / 10	Hypertension, ↑lipid	521	0.81	0.85	S	Members of diabetic association. Questionnaires mailed	OHA and Insulin	Multivariate logistic regression
Quah (12), 2011	Singapore/S’pore	63 / < 5	Hypertension, ↑lipid	699	0.91	0.91	S	T2DM fr public primary care polyclinics. Questionnaire at routine visit. systematic sampling	OHA/insulin/diet	Multiple regression analysis
Marrett (13), 2011	US/US	58 / 7.3	Angina, heart attack, stroke, peripheral vascular, heart failure	1984	0.81	0.85	S	Internet based survey with self-reported T2DM with OHA. Survey time point, randomly contacted	50% on OHA	Multiple linear regression
O’reilly (14), 2011	Canada/US	63.7 / 8.2	NR	1143	0.75	NR	S	Community dwelling T2DM, volunteered to be screened for RCT. Questionnaires mailed	323 (28%) on insulin	OLS Bootstrap standard errors
Lee (15), 2012	Korean/Korean	57.5 / NR	Hypertension, ↑lipid	1072; 858	NR	0.84	M	3 outpt clinic from three institute in diverse regions. Consecutive sampling: 1st, time of clinic visit; 2nd, follow up	OHA ± insulin	Backward elimination
Zhang (16), 2012	US/US	62 / 11.3	Hypertension, ↑lipid	7327	0.80	0.92	M	random sample fr health care plans. pts contacted and completed survey	Diet, OHA ± insulin	Multivariate linear regression
Luk (17), 2014	Hongkong/UK	59.2 / 8.9	Hypertension, ↑lipid	14,826	0.90	0.98	S	T2DM disease registry, referrals fr hospital and community clinics. 5 yrs data collection.	OHA ± insulin	Multivariate regression
Harris (18), 2014	Canada/Canada	55 / NR	NR	1696	0.71	0.82	S	French/ Eng; include T1 and 2, without diabetes. web based & gen pop survey	NR	Non-parametric bootstrapping
Kiadaliri (19), 2014	Sweden/UK	66.1 / 9	NR	1757	NR	0.88	M	National diabetes registry–from hospital, primary care, clinics. Survey during outpatient visits	diet as OHA ± insulin	OLS regression
Pan (20),2016	China/China	64.9 / < 10	55% with comorbidities	289	0.88	0.96	S	Tertiary hospital. Questionnaire after routine clinical examination	NR	BCA bootstrap and OLS
Hayes (21), 2016	Australia,Asia,EU,US/US, Poland,China	65.8 / 7.9	Macro and microvascular complication	11,140	0.82	0.83	M	ADVANCE trial patients. Longitudinal, at study entry, 2, 4 years, trial close out.	NR	Fixed-effect longitudinal regression
Jiao (22), 2017	Hongkong/Hongkong	64.84 /10-17	Hypertension, ↑lipid.	1275	0.86	0.88	M	T1&2 diabetics in outpatient clinic- hospital-based specialist clinic. Contacted within one month to fill questionnaire	NR	OLS with robust std errors.
Riandini (23), 2019	Singapore/Japan	60–64 /10-16	Hypertension, ↑lipid. Heart disease, PAD, arthritis	160	NR	0.77	M	T2DM	NR	Multivariable Regression
Pan (24), 2018	China/China	67.9/10.3–12.2	Hypertension	913	0.98	0.99	M	Community-based survey on T2DM-CDC health records. Detailed interview with questionnaire	NR	Generalised linear regression
Shao (25), 2019	US, Canada/Canada	62.6/10.55	Macro/ microvascular complication	8713	NR	0.72	S	ACCORD Trial pts: high risk CVD. Trial scheduled visits	NR	OLS/Fixed effects
Takahara (26), 2019	Japan/Japan	64 / 15	Musculo, dental, respi, gastro, urinary, cutaneous hearing impairmt	4963	0.90	0.94	M	13 centres, outpatient.	All meds	Multivariate regression model
Yfantoupoulos (27), 2019	Greece/UK	67.02 / 15.1	Hypertension, ↑lipid	938	0.71	0.82	M	One hospital centre+57 private centres. Interview	insulin users only	OLS regression
Zhang Yi, (28)2020	China/China	59.6 / 7.91	Hypertension, ↑lipid	7081	0.87	0.92	S	75 hospitals in 9 cities. Survey at clinics	OHA only	OLS regression
Pham (29), 2020	Vietnam/Vietnam	61.5/ 7	Hypertension	214	0.94	1.00	M	Police and general public. Hospital outpatient visits	NR	Tobit regression
Chao (30), 2020	China/China	59.5 /1 0.5	Hypertension, ↑lipid, complications	12,583	0.94	0.99	M	BEYOND II trial pts. hospital-based, multi-center cohort study. Physician administered questionnaires	All on insulin. +/- OHA;	OLS regression
Chen (31), 2021	China/China	60.1 / 10.1	Macro/microvascular complications	507	0.88	0.95	S	Outpatient in endocrine, nephron clinic, dialysis centre.	OHA ± insulin, diet	OLS regression
Kuo (32), 2021	Taiwan/Taiwan	57.48 / 4.54	Hypertension, ↑lipid, liver disease, depression, cancer, copd, connective tissue disease	2104	0.84	0.98	M	National Health Interview Survey (NHIS) 2009–2013 + national Health Insurance Research Database (NHIRD) 2002–2013. Survey data from outpatient, inpatient, emergency medical records.	OHA ± insulin, diet	Multivariable ordinary least sq regression models (OLS)
Laxy (33), 2021	Germany/Germany	72.5 / 11.5	Hypertension, cancer, asthma, chronic bronchitis	8755	0.78	NR	S	Population based cross sectional (2016). Postal survey/telephone interviews	NR	Linear regression
Neuwahl (34), 2021	US/US	59 / 6.8	14.0% had a history of cardiovascular disease	5103	0.79	NR	M	LOOK-AHEAD trial: overweight and obese HUI-3 at baseline, 3, 6, 9, and 12 months; every 6 months through 10 years; and once during years 10–13.	15.4% on insulin	Fixed effect model
Keng (35), 2022	UK/UK	62.8 / 9.7	Hypertension	11,683	NR	0.906	M	ASCEND trial patients- with DM, without CV disease. Questionnaires sent at end of 7-year study.	Ace-inhibitors, statin	OLS regression

### 2.5 Data analysis and synthesis methods

Published HSUV for all stages and severity of T2DM complications were extracted. When presented with multiple HSUVs, marginal utility reported in comparison with the absence of complication were preferred over disutilities alone. HSUV were elicited using standard conversion algorithms. Regression analysis were used to estimate decrements while adjusting for confounding variables such as, clinical characteristics (duration of diabetes, age onset of diabetes, body mass index, treatment, urine protein, HbA1C), diabetes related complications (stroke, heart disease, foot ulcers, amputation, neuropathy, nephropathy, retinopathy) and presence of comorbidities (hypertension, hyperlipidaemia, depression, cancer, chronic bronchitis, asthma). Only adjusted HSUV decrement estimates from statistical models were extracted, along with the uncertainty represented by 95% confidence intervals or standard errors. Whenever HSUVs were reported from multiple statistical models, the best fitting model preferred by the authors were extracted.

Since experience using formal synthesis methods is limited for HSUVs with high degree of heterogeneity in valuation methods and patient population, this review was conducted without meta-analysis. Synthesis methods were by vote-counting based on direction of effect. Informal methods to investigate heterogeneity included ordering tables by methodology characteristics such as study characteristics (country, tariff, ethnic, study design), population characteristics (inclusion/exclusion criteria, age, HbA1c, comorbidities), complications and timing of HSUV collection etc.

Complications were grouped into 2 subsets: macrovascular and microvascular, as well as individual complication types and severity for overview comparison. HSUV decrements were extracted for individual complication and stratified according to severity whenever possible (S2 Table). Cardiovascular complications in diabetes involves premature atherosclerosis [19]. Angina occurs when the coronary artery’s ability to supply adequate amount of oxygenated blood is insufficient for increased cardiac demand. Myocardial infarction (MI) occurs during ischemia or acute thrombosis due to atherosclerotic plaque rupture. Hence, angina was categorized as mild cardiovascular complication and MI the severe form [19]. Mild stroke include transient ischemic attack (TIA) with symptoms lasting from minutes to less than 24 hours; severe stroke included ischemic stroke caused by infarction, with symptoms lasting more than 24 hours [20].

All stages of diabetic kidney disease (persistent albuminuria, persistently reduced estimated glomerular filtration rate [eGFR <60 ml/min per 1.73 m^2^], or both, patients with a kidney transplant, on hemodialysis or peritoneal dialysis) were captured from the included studies [21]. We categorized early symptoms of albuminuria as mild nephropathy; ESRF or dialysis as severe nephropathy. Broadly defined nephropathy included those with eGFR >10mL/min to <60mL/min. Some authors defined retinopathy severity by the international clinical diabetic retinopathy disease severity scale (AAO), such as mild, moderate, severe Non-Proliferative Diabetic Retinopathy (NPDR), PDR [22, 23]. For our synthesis, mild retinopathy included NPDR stages while moderate stage included PDR [24]. Severe retinopathy included any sight-threatening stages or blindness. For studies measuring HRQoL using visual acuity (VA), severity stratification was based on author’s reported staging and definition.

Mild diabetic neuropathy commonly presents with numbness while severe forms are when patients are unable to heel-walk. After reviewing the included studies, we categorized severe neuropathy where pain symptoms were present [25]. We grouped foot ulcer as the mild stage of limb complication, and amputation as the severe stage based on the Wagner system which assesses foot ulcer depth and the presence of osteomyelitis or gangrene [26].

Although severe hypoglycemia is often defined when patients are unable to self-treat and needing the assistance of others, its definition varies greatly from the included studies [27–30]. We classified them into mild, moderate and severe stages based on author’s definition from individual studies. Some studies defined severe hypoglycemia by increased hypoglycemia frequencies (n = 2 studies) [29, 31], needing assistance (n = 3 studies) [30, 32, 33], timing of hypoglycemia episodes—daytime or nocturnal (n = 2 studies) [12, 28], symptomatic or not (n = 3 studies) [34–36]. 3 studies stratified severity into 4 levels while the rest did not.

Where complications were broadly defined by an umbrella term, HSUVs were extracted as it is and categorized as ‘undefined’, without being able to stratify into different levels of severity. Ranges of HSUV decrements across macrovascular and microvascular complications, as well as across each individual type of complications were compared. HSUV decrements from self-report complications and those obtained from medical records were compared. When longitudinal studies reported HSUV for event year when complication was experienced or as history before event year, results from fixed effect model were used to address the bias caused by time-invariant covariates [7, 34].

Quality of studies (risk of bias) were assessed using the modified elements of critical appraisal outlined by McMaster University and from National Institute for Health and Care Excellence (NICE) Technical Support Document [11, 37] (S3 File).

## Results

### 3.1 Study selection

**Fig 1:**
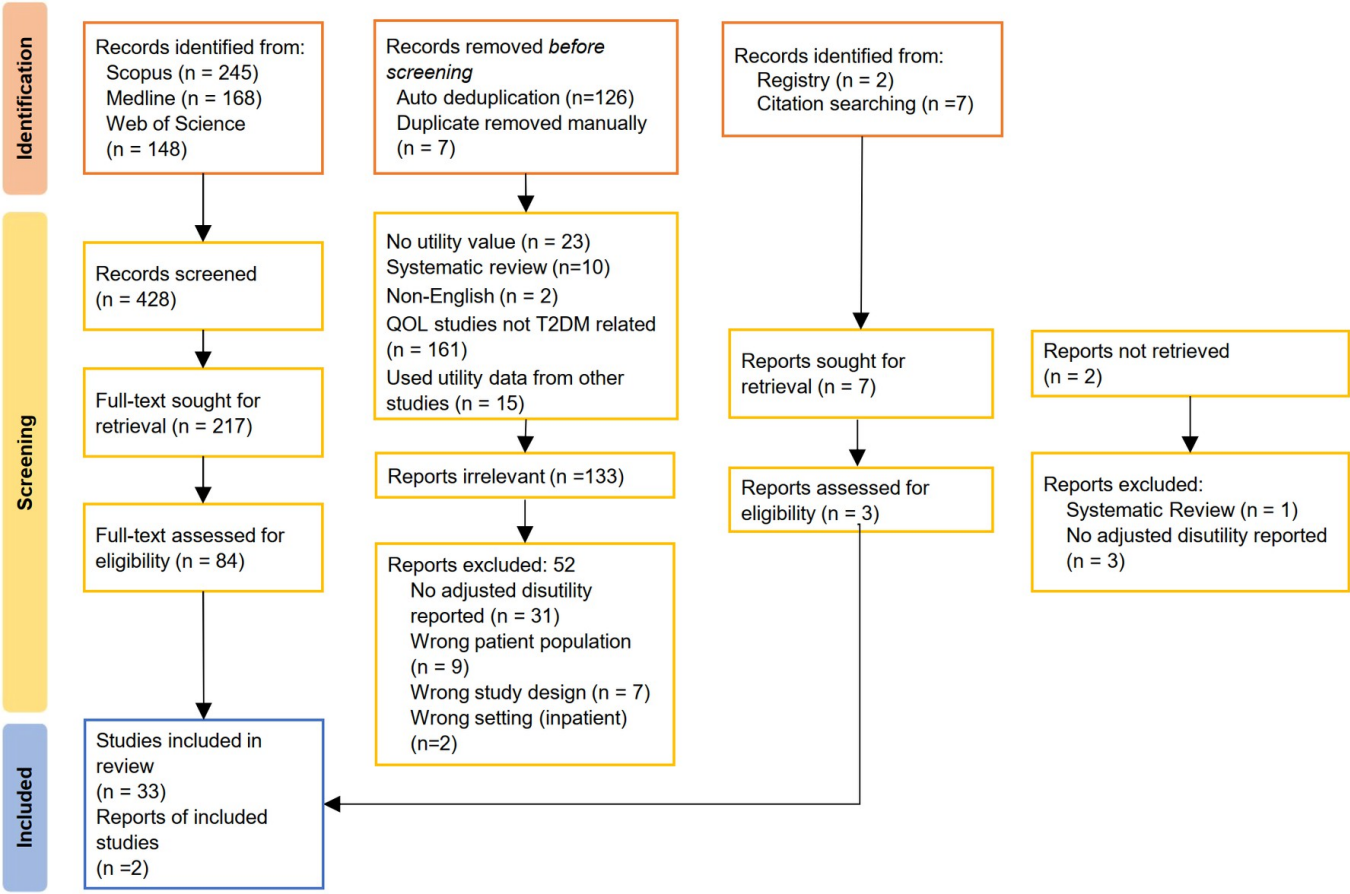
PRISMA flow diagram. *Note*: PRISMA: Preferred Reporting Items for Systematic Reviews and Meta-Analyses.

Initial searching yielded many results with generic terms for HRQoL such as ‘quality of life’ and ‘health state.’ Keywords were refined to be more specific to increase the relevance and efficiency of more focused search. For example, ‘quality of life’ terms had to be incorporated with ‘utility values or similar terms to exclude irrelevant studies which reported quality of life without reporting utility values. After removing duplicates, a total of 428 primary utility-elicitation studies were identified from multiple, iterative searching [14], where 35 studies were included for the review (Fig 1). A total of 52 studies were excluded: 31 reported utility values without adjustments for co-variates, 9 were of wrong patient population (For example, utility values for kidney transplant patients or myocardial infarction patients compared between those with and without diabetes) 7 were of wrong study design (for example, RCTs compared between interventions) and 2 were of inpatient setting.

### 3.2 Study characteristics

Table 1 summarizes background information about the included studies for the past two decades, including the study country, country tariff adopted for the study, adjusted comorbidities and statistical methods applied. For the duration of 2000–2010, studies were mainly from Western countries such as United Kingdom (n = 4) [7, 38–40], United States (n = 4) [29, 41–43], Canada (n = 1) [44] and Norway (n = 1) [45] with only one from an Asian country (Taiwan) [22]. From the year 2011 onwards, there were more publications by Asian countries such as Hong Kong (n = 2) [31, 46], China (n = 4) [24, 35, 47, 48], Taiwan (n = 2) [36, 49], Korea (n = 1) [9], Japan (n = 1) [12], Singapore (n = 2) [50, 51], Vietnam (n = 1) [52] as well as other European countries such as Germany (n = 1) [53] and Greece (n = 1) [32], Canada (n = 3) [28], U.S (n = 3) [6, 34, 54], Sweden (n = 1) [55], U.K. (n = 1) [56], inter-region (n = 1) [57].

### 3.3 Patient characteristics

Mean age ranged from 60–70 years old; mean duration of diabetes from 19 studies consisted of patients with < 10 years diabetes, 14 studies consisted of patients with 10 or more years of diabetes and two did not report. The size of the study population ranged from 160 to 14,826. A total of six studies recruited patients from randomized controlled trial (RCT) while the remaining 29 studies were population-based study [24, 33, 34, 39, 56, 57].

One RCT oversampled overweight and obese patients while another RCT excluded patients with cardiovascular disease and impaired renal function [33, 56]; one RCT excluded patients with recent hospitalization for unstable angina and impaired renal function [34]; one excluded patients with recent events of cardiovascular disease and renal disease or amputation [33]. These may reduce the applicability of some results to all T2DM patients.

### 3.4 Reported outcomes

Although overall quality of the included studies fulfilled the NICE guidelines criteria, 7 did not detail if sample size was achieved, 8 studies did not state clear inclusion or exclusion criteria and response rates. 26 studies utilized appropriate tariff matching to the local setting, 5 were mismatched, 3 were unreported. 2 did not report uncertainty measurement (S3 Table).

All studies included macrovascular and microvascular complications except for eight single studies which reported HSUV for only one complication: retinopathy [22, 39, 40, 43, 48]; neuropathy [51]; hypoglycemia [28, 30]. One multinational study (ADVANCE) which included Asian countries, Europe, Australia and U.S utilized appropriate tariff for each region during the analysis [57].

In the recent years, studies reporting HSUV for complications included different severity of complication (e.g., TIA, stroke with sequelae instead of just *stroke*; MI, angina instead of just *coronary heart disease*; proteinurea, dialysis instead of just *nephropathy*) [12, 24, 41, 49, 56, 58]. Five studies measured HRQoL at multiple points: on year of complication occurrence (acute impact) and subsequent years after complication occurrence (historical impact) [7, 33, 34, 55, 57]. Complication details from 17 studies were self-reported by patients while 18 were retrieved from medical records, diagnosed by medical practitioners (S1 Fig).

**Fig 2:**
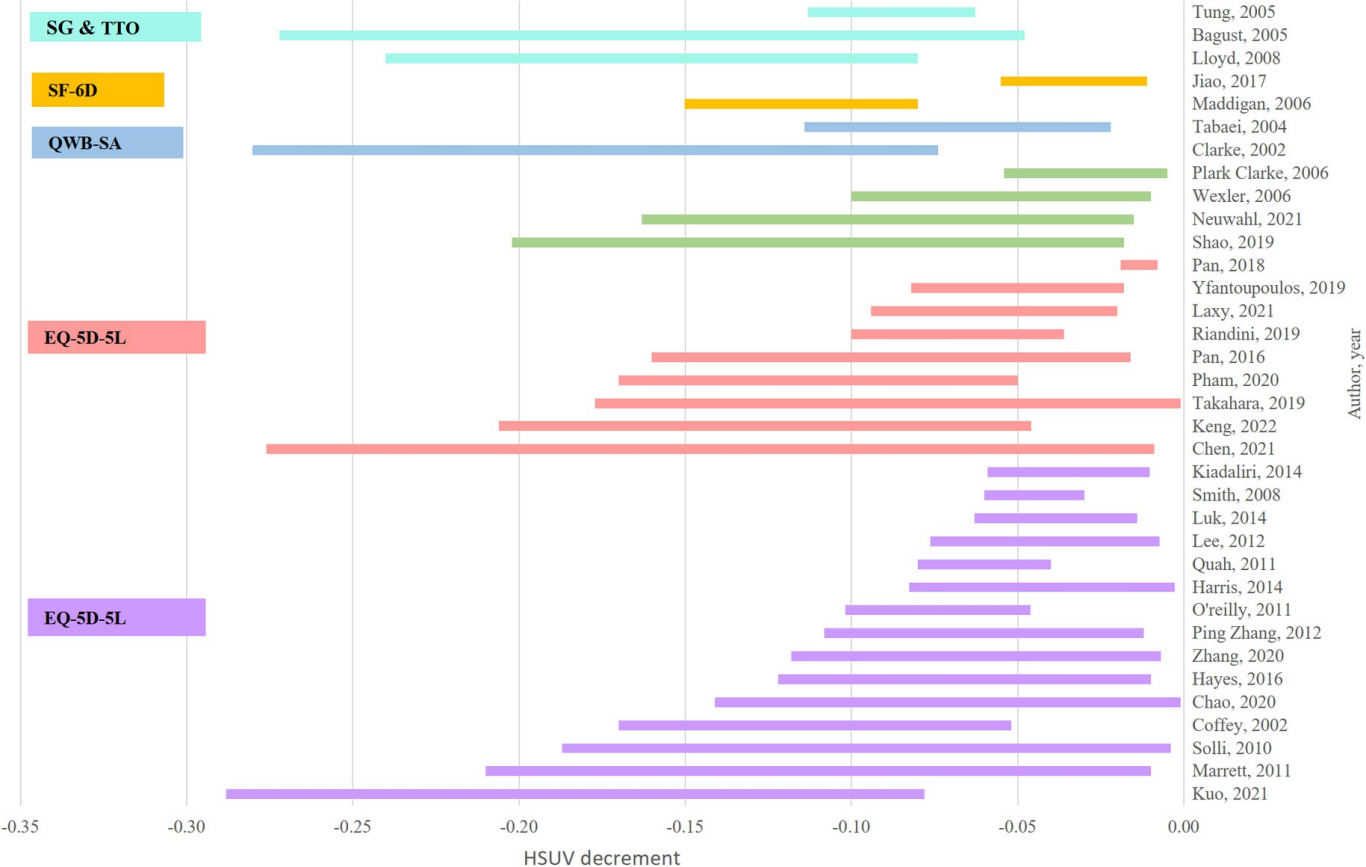
Overview of HSUV decrement range by valuation instruments. *Note*. standard gamble (SG); time-trade off (TTO); 5-level EQ-5D version, (ED-5D-5L); Short-form 6-dimensional (SF-6D); Self-administered Quality of Well-Being Index (QWB-SA); Health Utilities Index Mark 3 (HUI-3).

All studies utilized indirect or direct measures for HSUV valuation: 15 studies utilized EQ-5D-3L, nine EQ-5D-5L, four utilized HUI-3, two used QWB-SA, SF-6D and TTO each, and one used Standard Gamble. TTO range of HSUV decrements were larger compared to EQ-5D-3L, SF-6D and HUI-3 while EQ-5D-5L HSUV decrement range was larger compared to the EQ-5D-3L (Fig 2).

Considerable heterogeneity exists within studies due to difference in methods used (e.g., EQ-5D, SF-6D etc.), valuation methods (e.g., TTO, SG), tariffs used, inclusion and exclusion criteria of study population, and adjustments for different co-variates. These may result in a highly variable HSUV. In consideration of all these reasons, a formal synthesis may not be meaningful. Therefore, meta-analysis was not performed but range of HSUV decrements were reported instead.

### 3.5 Results of synthesis

Range of HSUV decrement were largest with cerebrovascular disease (stroke), retinopathy and amputation. Severe stages of stroke with sequelae, retinopathy with blindness and amputation were generally associated with largest HSUV decrements. Overall, there were no standardization of complication stage selected for HSUV elicitation. For example, ‘nephropathy’ could be defined in one study as eGFR < 30mL/min with proteinuria whereas in another study, stratified by many stages of severity: microalbuminaemia, eGFR 15-60mL/min, and end-stage renal failure (ESRF) or dialysis while some used an overall umbrella term [31, 35].

#### 3.5.1 Overview of HSUV decrement range by individual diabetes-related complication

HSUV decrement range (Fig 3) will be presented and discussed with details of each individual complication types (Figs 4–10).

**Fig 3:**
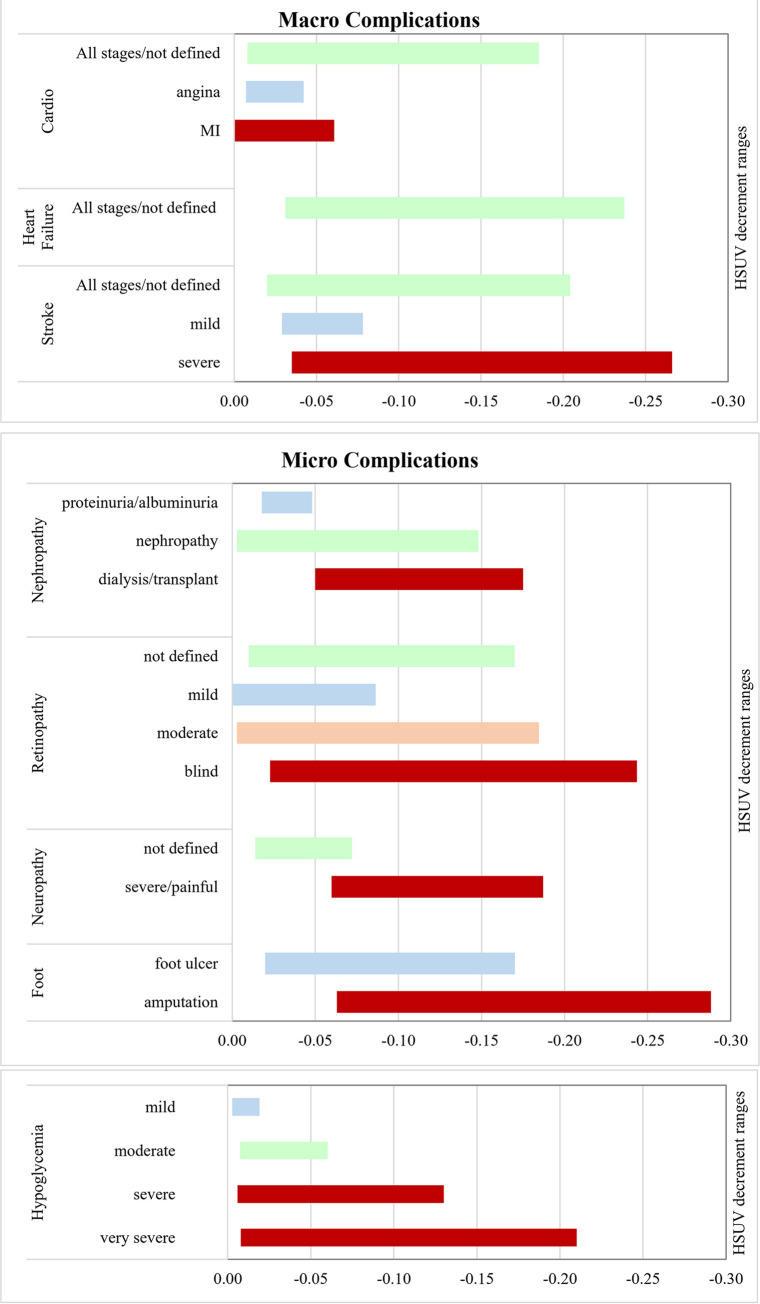
Overview of HSUV decrement ranges, by complication type. (A) macrovascular complications (B) microvascular complications (C) hypoglycemia.

**Fig 4:**
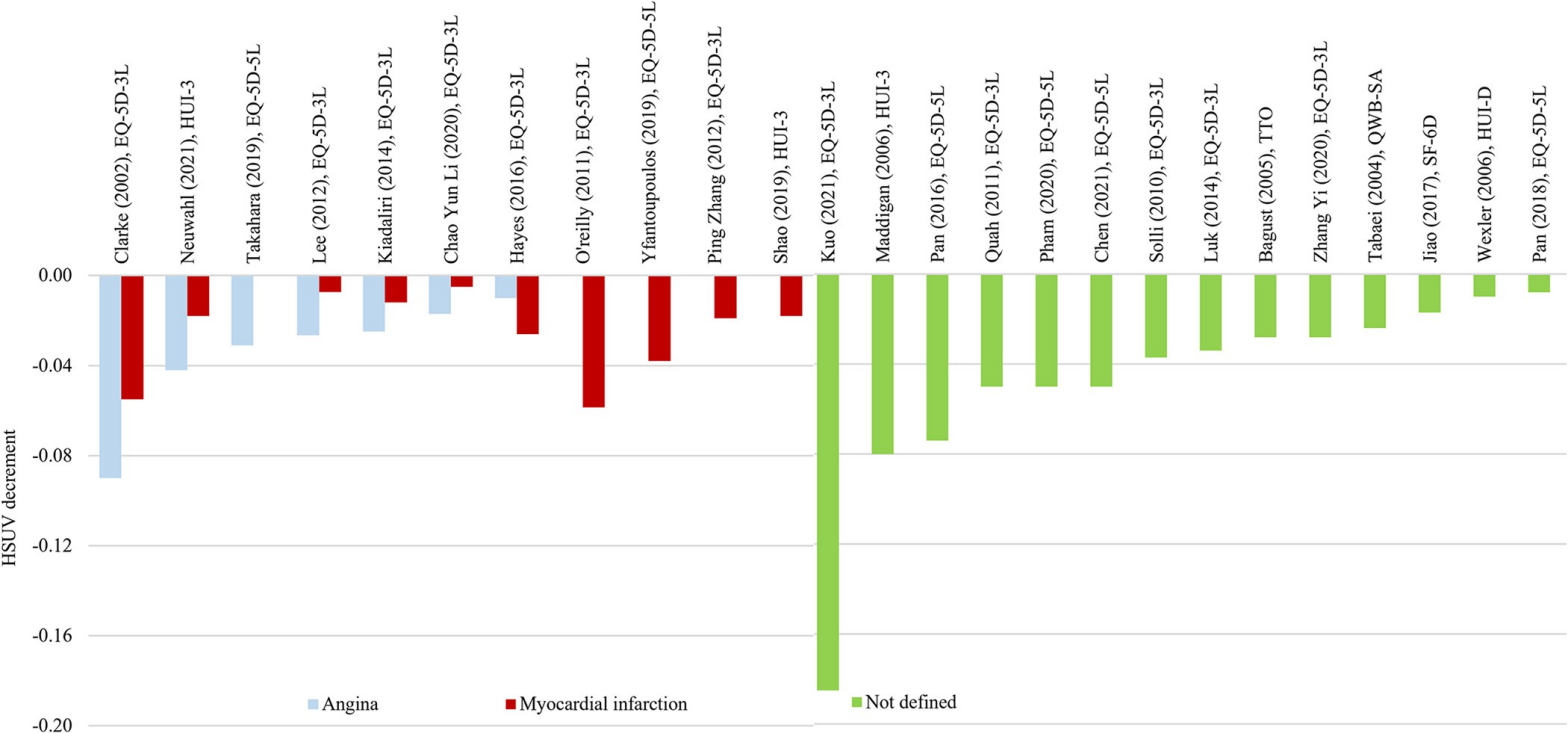
HSUV decrement values for cardiovascular complications, stratified according to different stages: Angina, myocardial infarction or undefined.

**Fig 5:**
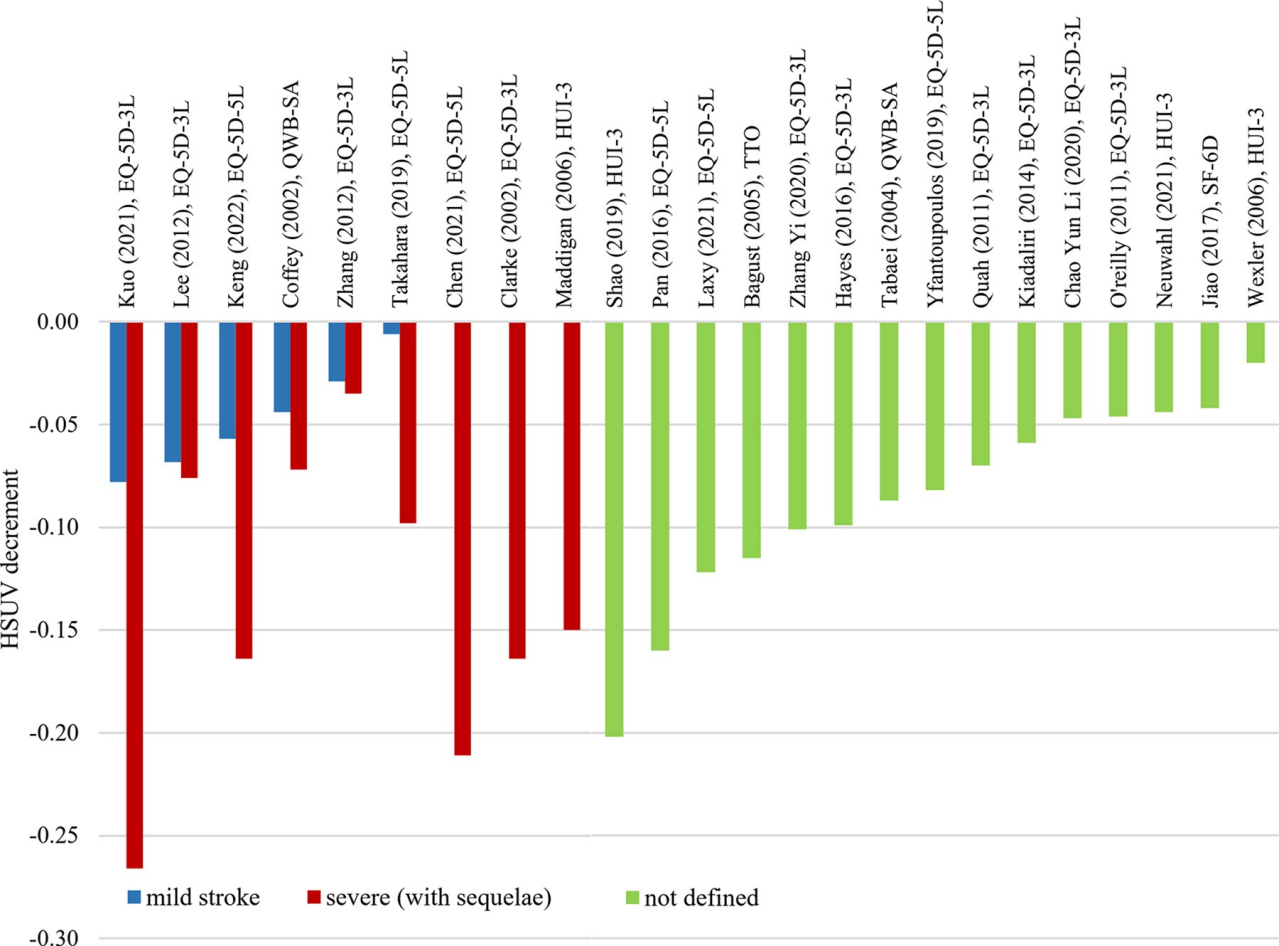
HSUV decrement values for cerebrovascular complications, stratified according to different stages: Mild stroke, severe stroke or undefined.

**Fig 6:**
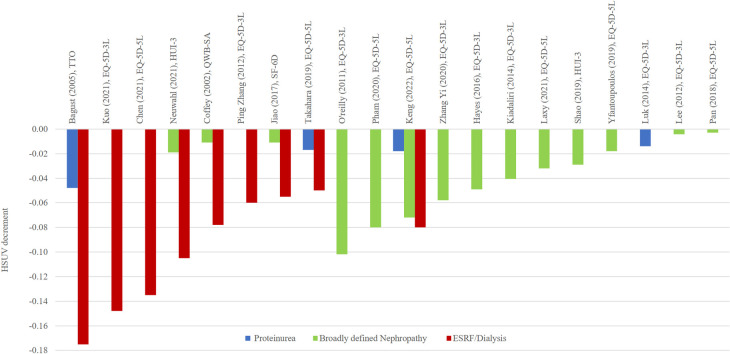
HSUV decrement values for diabetic nephropathy complications, stratified according to different stages: Proteinuria, broadly defined nephropathy or ESRF.

**Fig 7:**
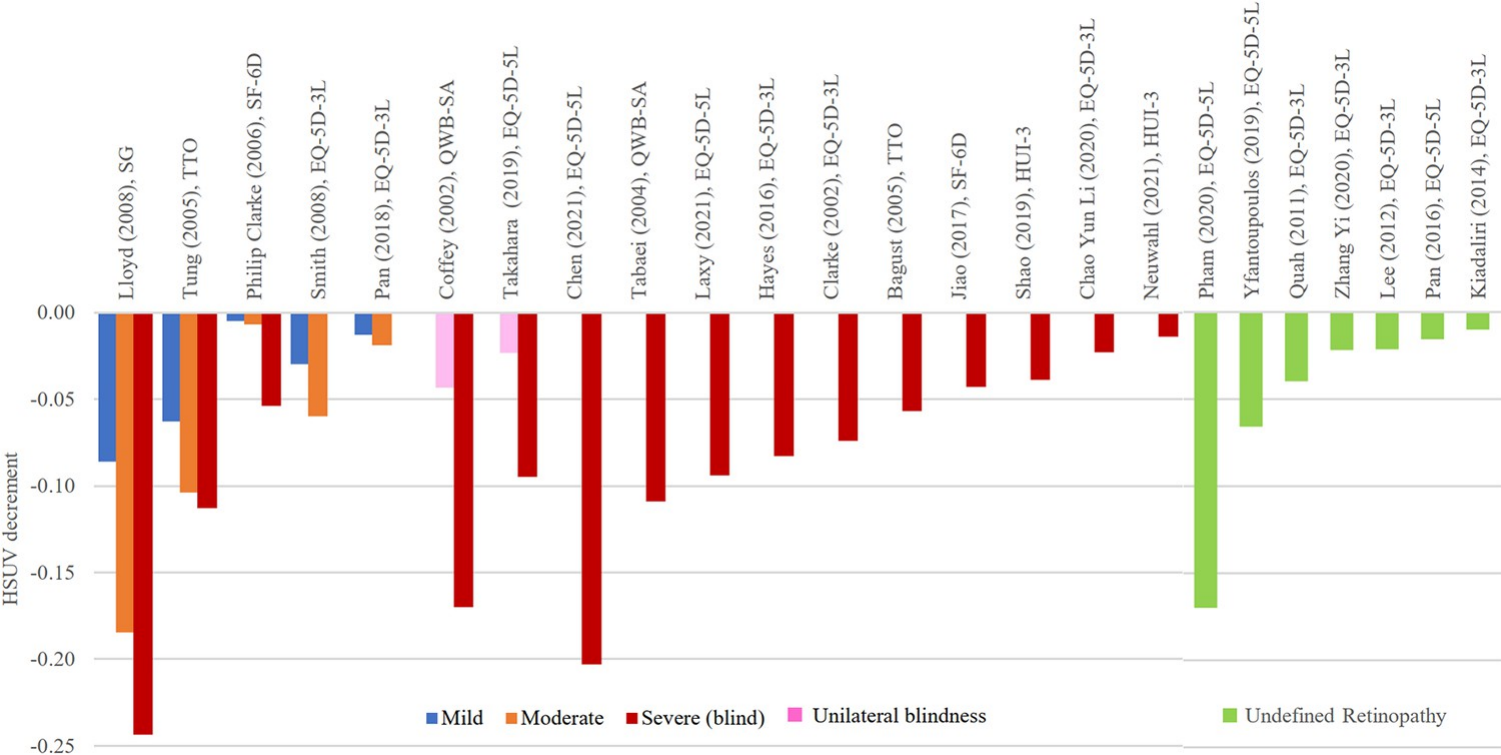
HSUV decrement values for diabetic retinopathy complications, stratified according to different stages: Mild, moderate, severe retinopathy, undefined retinopathy.

**Fig 8:**
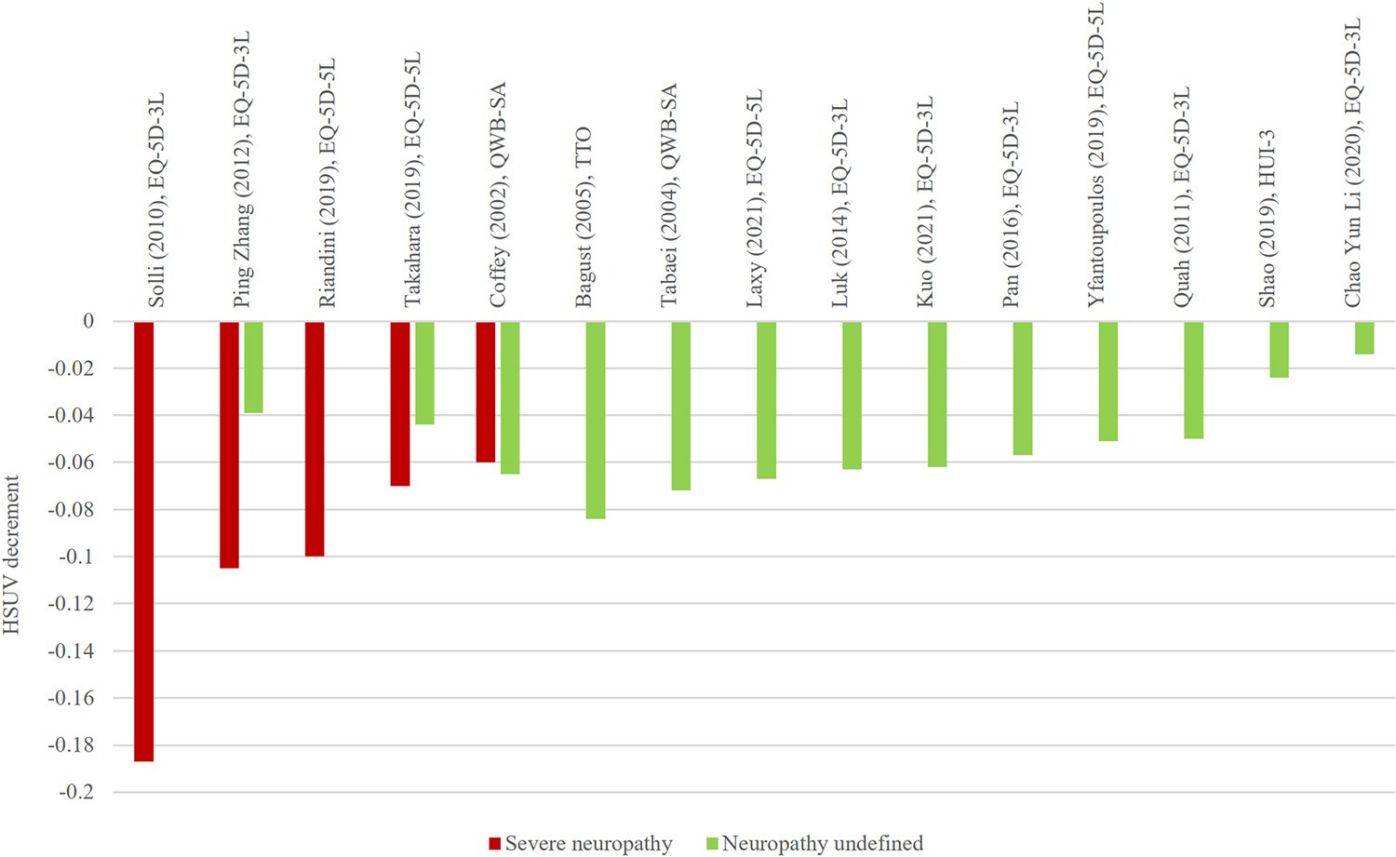
HSUV decrement values for severe neuropathy and undefined neuropathy.

**Fig 9:**
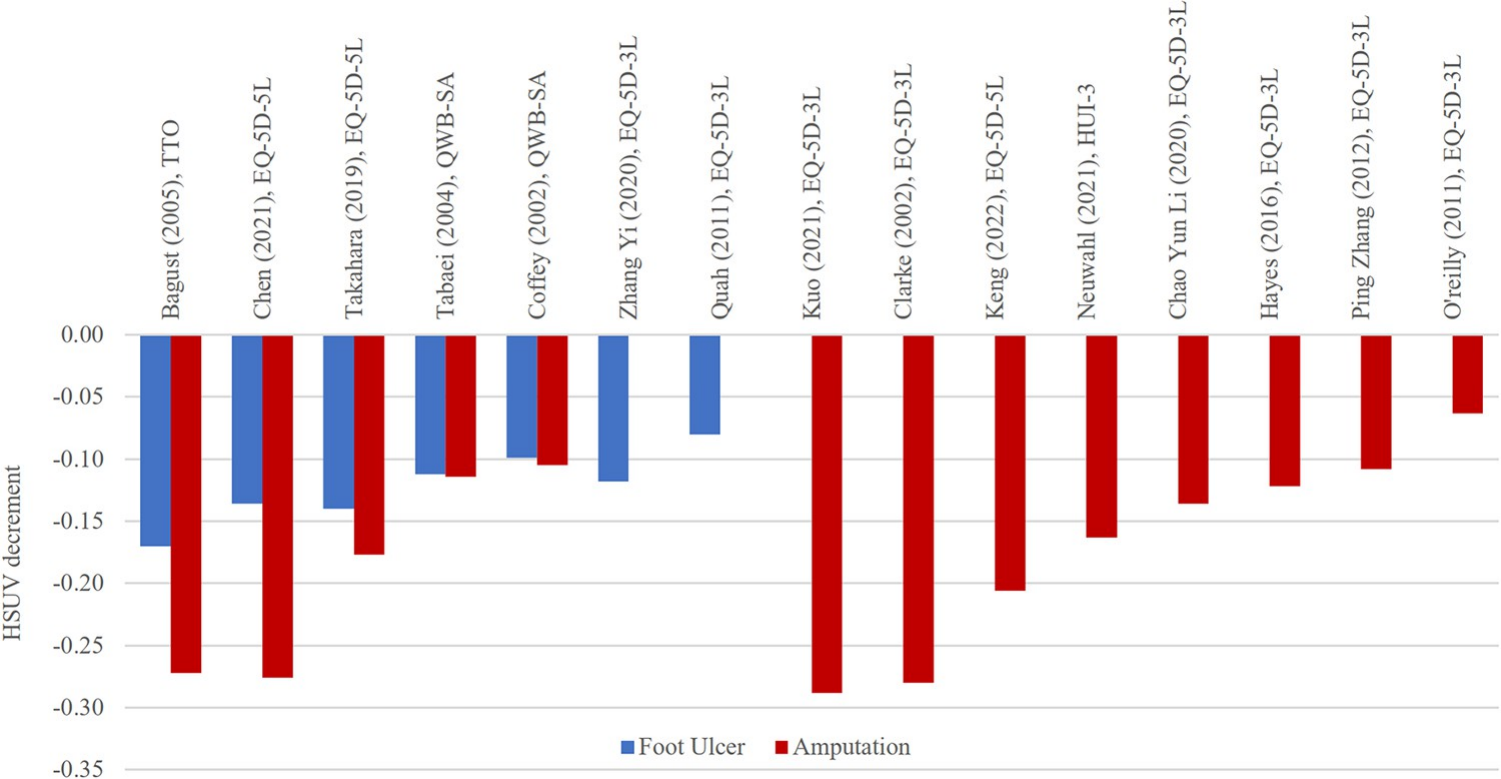
HSUV decrement values for foot ulcer and amputation complications.

**Fig 10:**
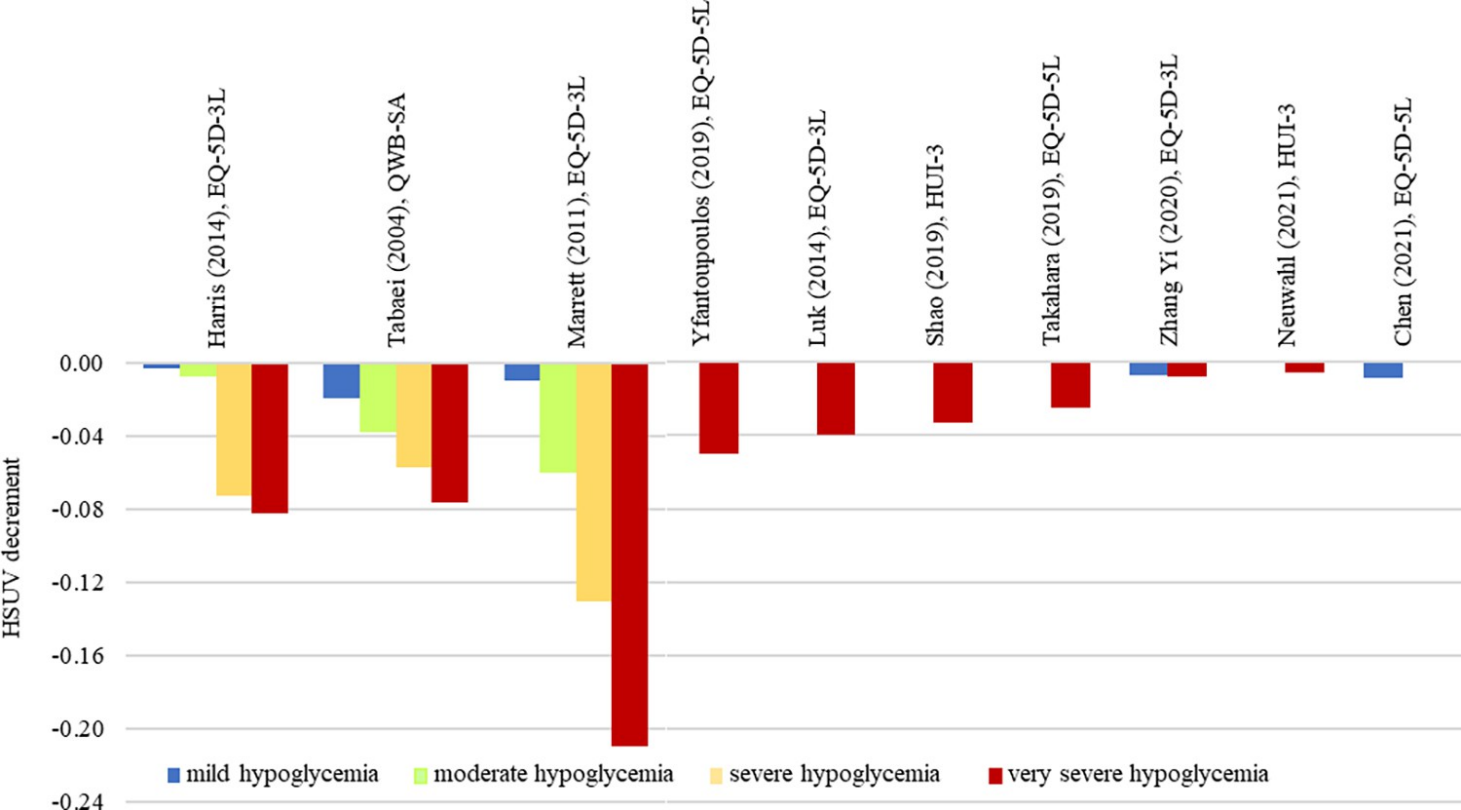
HSUV decrement values for mild, moderate, severe and very severe hypoglycemia.

*Cardiovascular complication (Fig 4, S4 Table).* When cardiovascular complications were all grouped together under one umbrella term (undefined), overall HSUV decrement range were wider (-0.008 to -0.185) compared to angina and MI reported separately. When angina and MI were both reported together in a single study, angina HSUV decrement were almost always larger than MI. Heart failure HSUV’s were mostly reported under one umbrella term without stratifying into disease severity (S2 Fig).

*Cerebrovascular (stroke) complication (Fig 5, S5 Table).* Range of HSUV decrement for mild stroke was -0.006 to -0.078 while severe stroke recorded a wider range of HSUV decrement from -0.035 to -0.266. When stroke was used as an umbrella term without further stratifying severity, HSUV decrement range was in between the mild and severe HSUV ranges (-0.020 to -0.202). HSUV decrement for intracranial hemorrhage stroke was only reported in one study [49].

*Nephropathy (Fig 6, S6 Table).* Only 4 studies included proteinuria HSUV decrement values, ranging from -0.017 to -0.048 [26, 30, 35, 54]. HSUV decrement for nephropathy was in the broad range of -0.003 to -0.102 [6, 47]. Severe stages saw a larger HSUV decrement range from -0.050 to -0.175. Whenever studies stratified severity by both nephropathy and ESRF, the latter health state would consistently have a larger HSUV decrement compared to broadly defined nephropathy stages [12, 33, 38, 41, 46]. Proteinuria stages showed the lowest HSUV decrement.

*Retinopathy (Fig 7, S7 Table).* Generally, when retinopathy was stratified into stages of severity, there was a stark difference between ranges of mild (-0.005 to -0.086), moderate (-0.003 to -0.185) and severe retinopathy (-0.023 to -0.243) HSUV decrements. Unilateral blindness had similar HSUV decrement ranges to moderate retinopathy while bilateral blindness had larger HSUV decrement. However, no clear difference was seen if compared between unilateral or bilateral diabetic retinopathy [47], or when visual acuity measured with the better eye [7]. The largest decrement value of severe retinopathy was -0.243 elicited using direct methods, TTO [40].

*Neuropathy (Fig 8, S8 Table).* Range of HSUV decrement for undefined neuropathy is narrow (-0.014 to -0.084) whereas severe stages had broader HSUV decrement range from -0.060 to -0.187. Where painful neuropathy was present, there was almost a doubling of the HSUV decrement. The largest reported HSUV decrement for neuropathy was -0.187 [45].

*Foot ulcer/amputation (Fig 9, S9 Table).* Foot ulcer HSUV decrement ranges (-0.080 to -0.170) share almost similar ranges with amputation HSUVs (-0.060 to -0.280) [12, 29, 36, 38, 35, 41]. Two studies [29, 41] showed almost similar HSUV decrements between foot ulcer and amputation whereas another 2 [36, 38] showed that amputation HSUV decrements were twice larger than those of foot ulcer.

*Hypoglycemia (Fig 10, S10 Table).* The HUSV decrement range for hypoglycemia almost doubled for each stage as it progressed from mild, moderate, severe to very severe hypoglycemia [28‒30]. However, unstratified hypoglycemia stages when used as an umbrella term produced narrow HSUV decrement range (Fig 3). Largest HSUV decrement was reported in an old study which only included patients on oral hypoglycemic agents [30].

## Discussion

**Fig 11:**
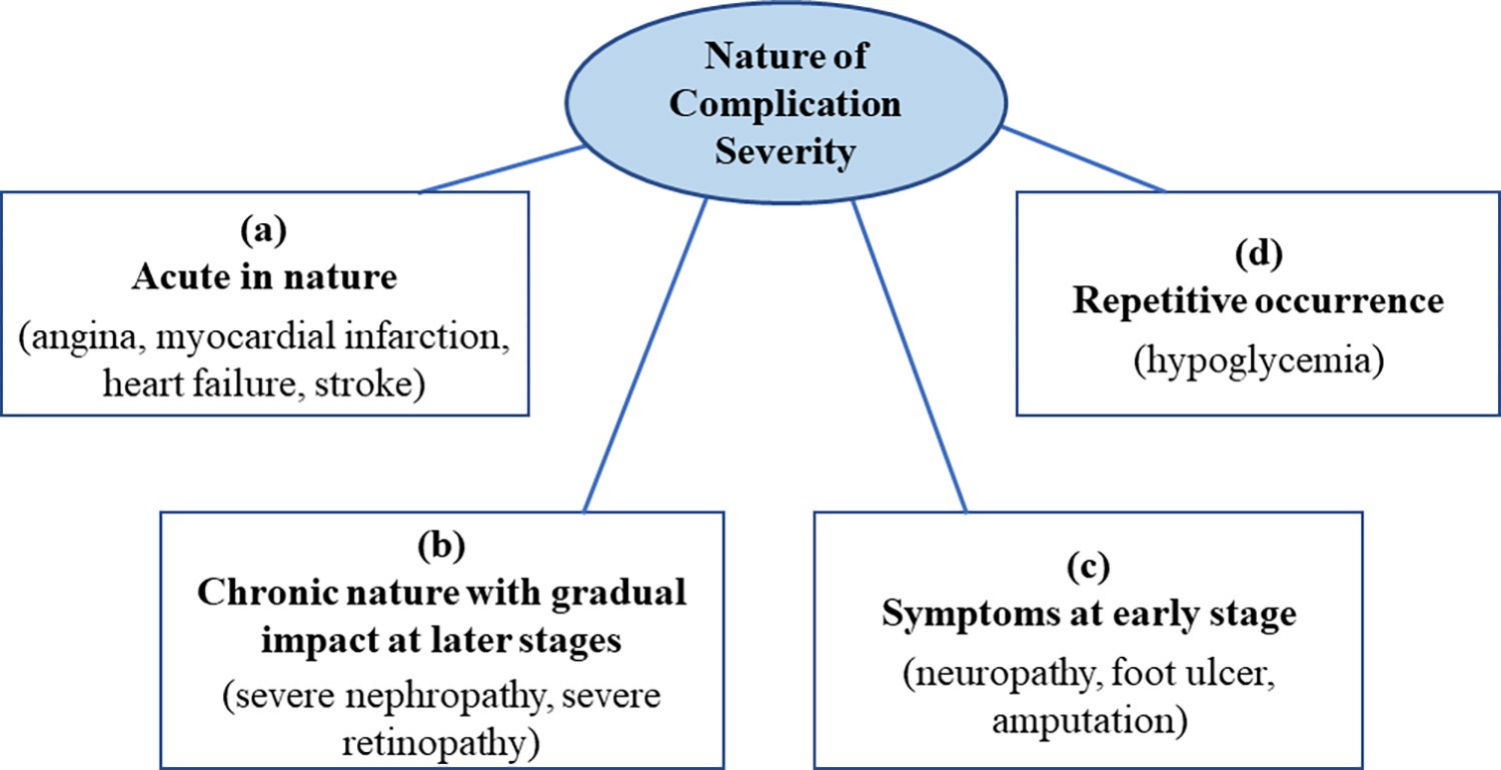
Different nature of complication severity which may affect HSUV decrement ranges.

Overall, the findings from this review showed that milder stages of complications often showed smaller HSUV decrements compared to severe stages. Definition across complication types and stages vary widely in HSUV studies. Different nature of complication severity: those with acute onset, those with chronic or lasting effects, those with symptoms at early stage, or those with repetitive frequency or episodes may have impacted the range of HSUV for each complication. Chronic complications with lasting impact such as amputation, severe stroke with sequelae, severe retinopathy with blindness were generally associated with largest HSUV decrements (Fig 11).

### 4.1 Nature of complication severity

#### a) Severity of complications: acute in nature

Acute clinical events may be associated with large HSUV decrement due to high levels of pain and discomfort during the event.

*Cardiovascular*. Generally, HSUV decrement range for both angina and myocardial infarction (MI) were small with most ranging between -0.06 to -0.018. One recent large UK study (n = 11,683) did not find any acute impact of MI on HSUV decrement observed in other studies [49]. Although MI episodes are usually more severe (life-threatening) compared to angina, the latter seemed to generate larger HSUV decrements whenever reported together in a single study. One previous systematic review for T2DM patients with MI reported similar HSUV decrements after adjusting for confounding variables (-0.019 to -0.065) [59]. Compared to acute management of MI, repeated angina episodes do not require urgent hospitalization or revascularization procedure. Therefore, the acute impact of MI may not be captured as the scope of this review covers only outpatient setting. Pooled analysis from Mok et al reported similar trends where HSUV decrement for angina (-0.017, 95% CI -0.036 to 0.022) were larger compared to MI (-0.007, 95% CI -0.014 to 0.007) [4].

Larger HSUV decrement ranges are reported when cardiovascular complications are defined under broad umbrella term such as ‘heart disease’, ‘ischemic heart disease’, ‘coronary heart disease’ encompassing acute clinical events of angina, myocardial infarction and heart failure. Currently, no consensus exists on how to define HSUV changes within these acute events therefore discrete event simulation models have been recommended by using discrete health status to capture these changes [14]. Severity or stages within cardiovascular complications should be stratified without generalization into one broad term.

*Heart failure*. Staging of heart failure severity by New York Heart Association (NYHA) classification system was absent in most HSUV elicitation studies, where they were classified under one umbrella term. Those with largest HSUV decrement values for heart failure were from a mixture of population-based studies and RCTs, two from UK and three from Taiwan and China [7, 24, 36, 49, 56]. One UK study reported large HSUV decrement values for acute phase of heart failure (in the event year) [56]. We could not synthesize much data due to the lack of disease severity stratification details.

*Cerebrovascular (stroke)*. In a meta-analysis by Mok et al, the range of HSUV decrement for stroke were -0.101 to -0.006 [4]. The authors selected only the largest marginal decrement for complications which reported multiple disutility value whereas the current review extracts all reported HSUV decrement values to stratify into different severity levels. Their upper range of HSUV decrement was much lower than the current review findings for overall stroke (-0.250) while the lower value coincided with mild stroke (-0.006). This goes to show that larger HSUV decrement for severe stroke may not be captured if severity is not stratified whereas HSUV decrement for mild stroke can be captured by the umbrella term. Transient ischemic attack (mild stroke) does not cause debilitating residual disability such as those from severe stroke thus exerting a lower HSUV decrement.

Longitudinal studies included in our review found a difference of HSUV decrement in ‘event year’ effect compared to ‘history of’ effect [33, 34, 55]. Recent literature has shown that complications with acute discrete events such as stroke or angina may impact HSUV decrement [14]. Nevertheless, the effect of timeline goes beyond the scope of the current study.

#### b) Severity of complication: Defined by chronic nature with gradual impact at later stages

Some complications do not seem to significantly affect HRQoL at earlier stages but gradually worsen as disease progresses to severe stages, such as nephropathy and retinopathy [60].

*Nephropathy*. Nephropathy definition between studies were found to vary widely, with different severity stages chosen to elicitate HSUV. Almost all diabetic patients typically progress from initial diagnosis to microalbuminuria, macroalbuminuria and elevated plasma creatinine (</+ 175 mmol/L) or ESRF [61]. Though microalbuminuria is the first sign of diabetic nephropathy, the smaller HSUV decrement seen could be due to unawareness to this mild, reversible, asymptomatic stage of the complication [46]. One Diabetic Kidney Disease (DKD) utility value review reported lower HSUV decrement of -0.011 and -0.047 for nephropathy stage [62]. Similarly, Mok et al used pooled estimates from meta-analyses, and reported a very narrow range for nephropathy (-0.080 to -0.020).

The larger upper range of HSUV decrements for ESRF (-0.050 to -0.175) compared to Mok’s narrower range (-0.055 to -0.050) could be due to sample size. (Mok et al, n = 2, versus n = 8 in the current review). As nephropathy progresses to advanced stages, patients were more likely to have poor HRQoL particularly in mobility and usual activity dimensions [63]. Previous HSUV reviews for DKD highlighted the need for DKD health states differentiation and not just the severe ESRF stage. The current review fills the knowledge gap of pre-ESRF HSUV decrement ranges (proteinuria and nephropathy stages) [10, 62].

*Diabetic Retinopathy (DR)*. The current review findings showed that there were marked inconsistencies with methods of reporting HSUV for diabetic retinopathy. Considerable heterogeneity exists in definition and measurement of retinopathy stages. Previous economic evaluation models have defined DR in 4 stages, absent (NoDR), non-sight-threatening (Non-STDR), sight-threatening (STDR), and bilateral blindness (BB) [64]. In the current review, most studies presented HSUV for blindness, the most severe stage of retinopathy. Even so, decrements were small, -0.1 and below. Previous reviews have found small changes in HSUV decrements across mild to severe retinopathy stages [65]. When broad umbrella term was used, very small HSUV decrement (< -0.07) were reported. One exception was a Vietnamese study where HSUV decrement was up to -0.17 but non-significant due to small sample size (n = 10) [52]. Early stages of retinopathy do not threaten sight and thus have little impact as daily activities can be performed without much interruption. This may explain the reason why majority of the HSUV studies selected the most severe stage i.e. blindness for retinopathy utility elicitation [66].

Generic EQ-5D or SF-6D estimates were found to be largely unresponsive to visual conditions possibly due to the lack of vision specific dimension [67]. The largest decrement differences between each retinopathy stage was reported when using direct method, SG [40]. This was consistent with another systematic review on DR where HSUV decrement estimates using SG were larger than those elicited using indirect methods (EQ-5D or SF-6D) [65]. Since SG is a direct utility elicitation method, the burden of visual function loss would be more accurately captured from patients experiencing vision loss themselves compared to indirect, societal weighted utility measures.

Another challenge for DR HSUV elicitation lies on unstandardized anchors in retinopathy studies. Although the current review did not find much difference, one systematic review on DR reported small decrements when HSUV decrement was anchored to Worse Seeing Eye (WSE), as compared when anchored to Best Seeing Eye (BSE). The updated NICE guideline addressed similar issues and recommended other condition-specific preference-based measure or direct valuation of own health for retinopathy HSUVs [68].

#### c) Severity defined by symptoms at earlier stages

The following microvascular complications related to the lower limbs are peripheral neuropathy, foot ulcer and amputation.

*Neuropathy*. Mild neuropathy showed lower range of HSUV decrements while severe neuropathy with pain showed larger HSUV decrements. Majority of studies did not stratify neuropathy into severity levels and grouped it under one umbrella term [24, 29, 31, 32, 34, 38, 45, 49–51, 53]. From the current review findings, consistent range of HSUV decrements were reported even in undefined neuropathy. It was interesting to note the contrast with nephropathy and retinopathy where there were inconsistent range of HSUV decrements in its ‘undefined or broadly defined stages.’ Early peripheral neuropathy showed considerable HSUV decrements in aspects of physical domain, even before significant pain was evident [69]. Since neuropathy is symptomatic even at its early stages, HSUV would be assumed to be affected by mild symptoms such as numbness or tingling. One Japanese and U.S study stratified symptomatic neuropathy as the mild stage, and painful neuropathy as the severe stage [12, 54]. Although similar EQ-5D were utilized in studies reporting severe neuropathy, HSUV decrement in a Norwegian study appear much larger compared to the rest [45]. One possible reason could be the fact that neuropathy complication was self-reported by patients while the rest were extracted from medical records. Since neuropathy is marked by symptoms, patients experiencing constant discomfort may overestimate and report larger HSUV decrements [38].

*Foot ulcer/ amputation*. The current review findings were consistent with many other previous reviews[3, 4, 33, 36, 38, 53, 54]. In all studies which reported amputation HSUVs with other complications, the lower limb complication consistently exerted largest HSUV decrement across multiple studies. Although amputation often had smallest sample size compared to other diabetes related complications, generated HSUV decrement was the largest (-0.177, 95% CI -0.291 to -0.063), (-0.280, 95% CI -0.389 to -0.170) [3, 4]. Many authors selected only amputation stages when eliciting HSUV for diabetic foot complication as foot ulcer would precede amputation in most cases [39, 49, 56].

We observed that HSUV decrements for foot ulcer and amputation were almost similar in some studies, implying that both foot-related complications though at different stages, may exert similar HSUV impact. Interestingly, a Thai study has shown that foot ulcer HSUV decrements were larger than amputation, especially among those who had weight-bearing problems and those with ischemic diabetic foot [70]. They must still cope with daily dressing, pain from ischemia, frequent doctor visits and medical leaves. HSUV decrements for foot ulcer could be due to pain, severity of ulcers, location of ulcers and foot deformation [71]. Furthermore, the presence of diabetic foot ulcer can result in permanent disability and amputation related to infection.

A large prospective, observational study, Eurodiale (European Study Group on Diabetes and the Lower Extremity) conducted in 14 European centers, reported that minor amputation was not associated with a negative impact on HRQoL in patients with diabetic foot ulcers [72]. This may explain the similar HSUV decrement values for foot ulcer and amputation reported in two of our included studies [29, 41]. Hence it is important to clearly define types of amputation (minor or major amputation), foot ulcer stages (active or chronic) in utility elicitation studies in order not to over or under-estimate HSUVs for economic evaluation.

#### d) Severity defined by repetitive occurrence of attack

Although these are treatment-related adverse events for diabetes, hypoglycemia is often classified as a diabetes-related complication.

*Hypoglycemia*. One Malaysian study stratified hypoglycemia severity both by time (nocturnal or daytime) and frequency of attacks. The authors found that severe hypoglycemia (defined by increased frequency or nocturnal hypoglycemia) has greater HSUV decrement compared with non-severe hypoglycemia, consistent with the current review findings [27]. Although by clinical guideline standards, “severe hypoglycemia” is defined as <40 mg/dL [2.2 mmol/L], many variations of severe hypoglycemia definitions exist in utility studies [73]. Despite the small HSUV decrement range (< -0.05), it is able to cause a significant impact on HRQoL with -0.03 suggested as benchmark for minimum clinical important differences in utility for persons with diabetes [74].

Studies over the recent years have seen lower HSUV decrements for hypoglycemia complication. Comparing two studies which included only oral agents, Marrett (2011) reported HSUV decrement of -0.21 whereas Zhang (2020) reported HSUV decrement of -0.008. Hypoglycemia episodes increase with insulin secretagogues, which may explain the larger HSUV decrement in Marrett’s study where 50% of study subjects were on sulphonylureas [30, 54]. Zhang reported a 30-fold reduction of HSUV decrement possibly due to improvement of newer oral hypoglycemic agents over the last decade, with lesser hypoglycemia adverse reactions.

### 4.2 Comparison by year, region, study instruments, outcome reporting methods

It was interesting to note that majority of elicitation studies for the earlier 10 years (2002–2010) were published from European countries and the latter 10 years (2011–2021) seen an increase publication from Asian countries. We did not find a similar consistency with a previous review where East and Southeast Asian population yielded more conservative values of utility decrement [4]. The authors extracted European data from Beaudet [3], where HSUVs were selected only from two large studies performed 20 years ago [7, 38]. Improvements in healthcare technology to manage diabetes complications may have contributed to recent European studies having smaller decrement values [33, 34, 53].

In a 5-year longitudinal study conducted by Hayes et al with over 11,000 participants across 4 regions, Australasia, Asia, Europe and North America, there were no significant differences in the HSUV changes associated with incident complications in the fixed-effects longitudinal model, even though mean EQ-5D-3L utility scores differed depending on the value set used [57]. In other words, there is no effect of geographic region on quality-of-life changes association with complications as long as correct tariffs are used. Adjustment of HSUV over time to account for age decline may explain the indifference as it adjusts for mortality rates. Since it is recommended by ISPOR as best practice [14], this review only included studies with adjusted HSUV. Similarly, we did not find differences in HSUV ranges among different regions.

A variety of elicitation methods were used, indirect methods such as EQ-5D, HUI-3, QWB-SA, SF-6D questionnaires as well as direct methods namely SG and TTO. Two authors utilized different generic measures to compare HSUV decrements. A U.K. study revealed an equivalent decline of HSUV on both the EQ-5D and HUI-3 while a Korean study reported larger Spearman’s coefficient between the EQ-5D and the SF-6D scales occurring only in some HRQoL dimensions [9, 40]. However, no consistent pattern was found as all studies adjusted for confounder variables. When compared to three other studies utilizing direct methods, SG and TTO generally reported larger HSUV decrement ranges [22, 38, 40]. This was similar to Lung et al’s findings where TTO/SG scores were higher than studies using EQ-5D. Direct methods capture values that patients assign to their own health state, whereas the indirect measures such as EQ-5D or HUI-3 use published tariffs from societal views to assign patients’ descriptions of their health [75]. This may explain the difference observed.

HSUV decrement ranges from self-reported complications were narrower compared to those from medical records. Self-reported complications may be incomplete as patients could only be aware of certain symptomatic complications, not accounting for other non-symptomatic complications. However, advantages include possibly more accurate reflection on quality-of-life measures especially for symptomatic complications. A U.S study pointed out that HSUV for different stages of diabetes-related complications should focus on those that affect symptoms and functioning as self-reported data helps to assess patients’ awareness of complications which may affect quality of life [41]. On the contrary, retrieving medical records for complication histories produces more accurate clinical assessments of complications and its stages of severity. This can assist better understanding of complication severity impact on HSUV decrement ranges. Therefore, there seem no superiority of one method over the other.

## Strengths and limitations

Several limitations were encountered during the review. First, many studies did not define severity of complications and used only an umbrella term, rendering difficulty in categorizing some complication types to synthesize data for the purpose of the review. Second, although included studies reported measures of uncertainty by adjusting for sociodemographic and presence of multiple complications, the variable types were inconsistently reported across studies. This may not reflect the full range of uncertainty in the studies. Third, considerable heterogeneity was found across study population, country, study instruments, statistical methods for modelling and definition of individual complications. Therefore, pooling of estimates were not attempted but instead, range of HSUVs were reported to provide an overview for comparison. Despite those limitations, the strengths of the current study were that most included studies were from observational population-based studies, reducing the chance of bias or selective reporting. Another notable strength was, we attempted to classify individual complications by severity to provide uniformity for comparison of HSUV decrement ranges across the expected heterogenicity, allowing a broad overview for comparison.

## Conclusion

In conclusion, this systematic review compared the HSUV decrement ranges for different diabetes-related complications of different severity, elicited with direct and indirect methods. Severe stages of complications often present with larger HSUV decrement ranges compared to the milder stages. Understanding each individual complication and its nature of severity helps future researchers identify appropriate HSUV to populate economic models. The findings of this study provide informed outcomes to researchers in the presence of multiple heterogeneities (variation of study populations, countries, study instruments, statistical methods for modelling and definition of complication stages) when identifying HSUV for economic evaluation and policy analysis. Future studies could explore which health state would produce the most influential HSUV of each complication for cost effectiveness studies.

## Supporting information

S1 Checklist Prisma checklist. (PDF)

S1 Table Search strategies. (PDF)

S2 Table Summary of included studies sample size and severity groups. (PDF)

S3 Table Risk of bias assessment criteria. (PDF)

S4 Table HSUV decrement and definition for cardiovascular complication. (PDF)

S5 Table HSUV decrement and definition for stroke complication. (PDF)

S6 Table HSUV decrement and definition for nephropathy complication. (PDF)

S7 Table HSUV decrement and definition for retinopathy complication. (PDF)

S8 Table HSUV decrement and definition for neuropathy complication. (PDF)

S9 Table HSUV decrement and definition for foot ulcer and amputation complication. (PDF)

S10 Table HSUV decrement and definition for hypoglycemia complication. (PDF)

S1 File Abbreviation. (PDF)

S2 File Data extraction form items. (PDF)

S3 File Risk of bias assessment criteria. (PDF)

S4 File Risk of bias table. (PDF)

S1 Fig Overview of HSUV decrement ranges by reporting methods. (PDF)

S2 Fig HSUV decrement for heart failure complication. (PDF)
