# Supplementary material for: Health state utility values ranges across varying stages and severity of type 2 diabetes-related complications: A systematic review
Source: PLoS One. 2024 Apr 4;19(4):e0297589. doi: 10.1371/journal.pone.0297589 (PMC10994347; doi:10.1371/journal.pone.0297589)
Supplement: S1 Table — (PDF) [file pone.0297589.s002.pdf]

S1 Table : Search strategies

Search Strategies for Diabetes complication and utilities. (December 2021 – April 2022)

		Keywords and MESH terms		
	Database	Medline	Web of Science	Scopus
1	Diabetes	Type 2 diabetes/<br>T2DM<br>NIDDM<br>“Adult-onset diabetes”<br>“Non-Insulin Dependent”<br>“Non-insulin dependent diabetes mellitus”	Diabetes/<br>T2DM (All fields)	Diabetes/<br>T2DM
2	Hypoglycemia	hypoglyc$mia	Hypoglycemia OR hypoglycaemia	hypoglyc$mia
3	Coronary Heart Disease	Cardio* OR heart OR “coronary heart disease “Or “coronary artery disease” Or “isch$mic heart disease” Or “myocardial infarction”	Cardio* OR heart OR “coronary heart disease“ OR “coronary artery disease” OR "myocardial infarction" OR "isch$mic heart disease	Cardio* OR heart OR “coronary heart disease “Or “coronary artery disease” Or “isch$mic heart disease” Or “myocardial infarction”
4	Stroke	Stroke or cerebrovascular*	Stroke or cerebrovascular*	Stroke or cerebrovascular*
5	Nephropathy	Dialysis OR Nephro* OR renal OR kidney	Dialysis OR Nephropathy OR Diabetic kidney disease	dialysis OR nephro* OR renal OR kidney
6	Retinopathy	retinopathy	retinopathy	retinopathy
7	Neuropathy	Neuropathy OR “peripheral vascular”	Neuropathy OR “peripheral vascular”	Neuropathy OR “peripheral vascular”
8	Amputation & diabetic foot	Amputation OR foot OR peripheral	Amputation OR foot OR peripheral	amputation OR foot OR peripheral
9	Complication	Complication* OR microvascular OR macrovascular	Complication* OR microvascular OR macrovascular	Complication* OR microvascular OR macrovascular
10	Quality of life (OR)	Health-related quality of life<br>Quality of life<br>HRQOL<br>QOL<br>QALY<br>Quality-adjusted life year*<br>Quality adjusted life year	"Quality of life"<br>“health-related quality of life"<br>Quality adjusted life year	"Quality of life"<br>“health-related quality of life"<br>Quality adjusted life year
11	Utility (OR)	“Utility score”<br>“Utility value”<br>“Utility weight”<br>“Utility measure”<br>“Health utility*”	“Utility score*<br>“Utility value*<br>“Utility weight*<br>“Utility index”<br>“Utility measure”<br>“Health utility”	"Utility scores”<br>“Utility weight"<br>"Utility index"<br>"Utility value"<br>"Utility measure"<br>"Health utilit*"
