# Supplementary material for: Health state utility values ranges across varying stages and severity of type 2 diabetes-related complications: A systematic review
Source: PLoS One. 2024 Apr 4;19(4):e0297589. doi: 10.1371/journal.pone.0297589 (PMC10994347; doi:10.1371/journal.pone.0297589)
Supplement: S2 Table — (PDF) [file pone.0297589.s003.pdf]

S2(A) Table : Summary of Included studies : Individual Complication sample size and severity groups : -

● mild stage ● severe stage ● complications undefined

Author, Year, Sample size	Cardiovascu-lar	Heart Failure	Stroke	Nephropathy	Retinopathy	Foot ulcer/ amputation	Neuropathy	Hypo glycemia	Range of disutility values
Clarke, 2002<br>n = 3192	● n = 190<br>● n = 200	n = 66	● n = 69	-	● n = 101	● n = 19	-	-	0.074 - 0.280
Coffey, 2002<br>n = 2041		n = 141	● n = 107<br>● n = 57	● n = 199<br>● n = 37	● n = 129<br>● n = 135	● n = 92<br>● n = 37	● n = 192<br>● n = 376	-	0.052-0.170
Tabaei, 2004<br>n = 888	● n = 240	-	● n = 27		● n = 195	● n = 18<br>● n = 142	● n = 400	● n = 71	0.022-0.114
Bagust, 2005<br>n = 4641	● n = 1002	-	●n = 297	● n=NR<br>● n = 111	●n = 172	●● n = 362 foot ulcer and amputation	● n = 1160	-	0.048-0.272
Tung, 2005<br>n = 725	-	-	-	-	● n = 65<br>● n = 12<br>● n = 9	-	-	-	0.063-0.113
P. Clarke, 2006<br>n = 4051	-	-	-	-	● n = 4010<br>● n = 41	-	-	-	0.005-0.054
Maddigan, 2006<br>n = 5134	●n = 1088	-	●n = 267	-	-	-	-	-	0.08-0.15
Wexler, 2006<br>n = 909	● n = 316	n = 116	● n = 148	-	-		-	-	0.010-0.100
Smith, 2008<br>n = 2074	-	-	-	-	● n = 249<br>● n = 62	-	-	-	0.030-0.060
Lloyd, 2008<br>n = 319	-	-	-	-	● n = 91<br>● n =??<br>● n = 10	-	-	-	0.080-0.240
Solli, 2010<br>n = 356	● n = 65		● n = 19				● n = 17	● n = NR	0.004-0.187
Quah, 2011<br>n = 699	● n = 101	-	● n = 53	-	●n = 170	● n = 37	● n = 170	-	0.040-0.080
Marrett, 2011<br>n = 1172	-	-	-	-	-	-	-	●n = 977<br>● n = 195	0.010-0.210
O Reilly, 2011<br>n = 1143	● n = 157	-	● n = 82	● n = 28		● n = 14	-	-	0.046-0.102
Lee, 2012<br>n = 1072	● n = 87<br>● n = 50	n = 46	●n=18<br>● n = 60	● n = 39	● n = 253	-	-	-	0.007-0.076
Ping Zhang, 2012<br>n = 7327	● n = 1,529	n = 759	● n = 125<br>● n =519	● n = 69		● n = 168	●<br>n = 1,700<br>●<br>n = 1,969	-	0.012-0.108
Luk, 2014<br>n = 14,826	● n = 3202	-	-	● n = 6953			● n = 667	● n = 1245	0.014-0.063
Harris 2014<br>n = 1181	-	-	-	-	-	-	-	● n = 590<br>● n = 285	0.003-0.083
Kiadaliri. 2014<br>n = 1757	● n = 121<br>● n = 122	n = 150	● n = 221	● n = 94	● n = 75		-	-	0.010-0.059
Pan, 2016<br>n = 289	● n = 57	-	● n = 18	-	● n = 136	-	● n = 109	-	0.016-0.160
Hayes, 2016<br>n = 11,140	●n = 483<br>●n = 247	n = 270	● n = 335	● n = 89	● n = 44	● n = 39 amputation	-	-	0.010-0.122
Jiao, 2017<br>n = 1275	●n = 113	-	●n = 72	● n = 233<br>● n = 75	●n = 66 STDR	-	-	-	0.011-0.055
Riandini, 2018<br>n = 160	-	-	-	-	-	-	● n = 80		-0.100

Author, Year, Sample size	Cardiovascu-lar	Heart Failure	Stroke	Nephropathy	Retinopathy	Foot ulcer/ amputation	Neuropathy	Hypo glycemia	Range of disutility values
Pan, 2018<br>n = 913	● n = 211	-	-	● n = 113	● n = 56<br>● n = 102		-	-	0.008-0.019
Shao, 2019<br>n = 8713	● n = 1307	n = 355	● n = 481	● n = 44	●n = 171		● n = 301		0.018-0.202
Takahara, 2019<br>n = 4963	● n = 586	-	● n = 268<br>● n = 144	●n = 521<br>●n = 94	●n = 382<br>●n= 238	●n = 69<br>●n = 55	● n = 779<br>● n = 1891	● n = 179	0.001-0.177
Yfantopoulos, 2019<br>n = 938	● n = 236	n = 29	● n = 43	● n = 81	● n = 79		● n = 70	● n = 140	0.018-0.082
Zhang Yi, 2020<br>n = 9570	● n = 1296	-	● n = 590	●n = 777	●n = 1341	● n = 244		●n = NR<br>●n = NR	0.007-0.118
Pham, 2020<br>n = 214	● n = 56	-	-	●n = 18	●n = 10	-	-	-	0.050-0.170
Chao, 2020<br>n = 12,583	● n = 407<br>● n = 207	n = 84	● n = 443		●n = 650	● n = 183	● n = 3454	-	0.001-0.141
Chen, 2021<br>n = 507	● n = 85	n = 41	● n = 60	●n = 48	●n = 11	● n = 42<br>● n = 7	-	● n = 119	0.009-0.276
Kuo, 2021<br>n = 2104	● n = 464	n=94	●n=259<br>●n=53	●n=37		● n = 9	● n = 1,036	-	0.078-0.288
Laxy, 2021<br>n = 8785		n = 161	● n = 125	● n = 82	● n = 10		● n = 166	-	0.020-0.094
Neuwahl, 2021<br>n = 5103	● n = 327<br>● n =36	n = 36	● n = 138	● n = 250<br>● n = 5	● n = 148	● n = 20	-	●NR	0.015-0.163
Keng, 2022<br>n = 11,683		n = 105	●n = 214<br>●n = 22	● n = 854<br>● n = 584<br>● n = 42	-	● n = 60	-	-	0.046-0.206
