# Supplementary material for: Health state utility values ranges across varying stages and severity of type 2 diabetes-related complications: A systematic review
Source: PLoS One. 2024 Apr 4;19(4):e0297589. doi: 10.1371/journal.pone.0297589 (PMC10994347; doi:10.1371/journal.pone.0297589)
Supplement: S3 Table — (PDF) [file pone.0297589.s004.pdf]

S3 Table: Risk of bias assessment criteria

1.	Study Purpose	:	Was the purpose stated clearly
2.	Justification of study	:	Was relevant background literature reviewed?
3.	Design	:	Randomized Control Trial (RCT) /Cohort/cross-sectional
4.	Sample size/ Respondent selection	:	Was sample size described in detail-how was sampling done, how many, who, where? justified-ethic procedure done, informed consent? Is the study representative to the population from which they were recruited? if more than 1 group, similarity exist between groups?
5.	Inclusion/exclusion criteria	:	Does the study make clear the inclusion/exclusion criteria?
6.	Response rates to instrument used	:	Are response rates reported. If reported, rates unlikely to be a threat to validity? Drop outs reported/missing data? drop out size is small? Will the size of loss to follow up NOT likely affect validity of estimates? Loss to follow-up
7.	Appropriateness Tariff	:	Is the tariff reported and does it match study population
8.	Outcome measures	:	Is the measure used valid in the group of patients? were the objective, standard criteria used for measurement of the condition? if there were multiple outcomes, was that considered for statistical analysis?
9.	Uncertainty measurement		Is there any reported uncertainty measurement (e.g., 95% confidence interval, standard error)? Were confounding factors identified? Strategies to deal with confounding factors?
10.	Outcome measures		Were outcome measures reliable and valid?
11.	Results		Results significant? Analysis method appropriate? What was clinical importance of results?
12.	Conclusions and Implications		Conclusions were appropriate given study methods and results. what did study conclude? implications for practice? limitation or bias in study?

Adapted from : Papaioannou, Chiu, NICE, McMaster Uni

S3(A) Table: Risk of bias Table

Study ID	Purpose	Justification	Sample size	Inclusion/ exclusion	Response rates	Appropriate Tariff	Outcome measures	Uncertainty measurement	Results	Conclusions/ Implications	Total points
Clarke, 2002	Yes	Yes	Yes	Yes	Yes	Yes	Yes	Yes	Yes	Yes	**10/10**
Coffey, 2002	Yes	Yes	Yes	Yes	Yes	Unsure	Yes	Yes	Yes	Yes	**9/10**
Tabaei, 2004	Yes	Yes	Yes	No	No	Unsure	Yes	Yes	Yes	Yes	**7/10**
Bagust, 2005	Yes	Yes	Yes	Unsure	Unsure	Yes	Yes	Yes	Yes	Yes	**8/10**
Tung, 2005	Yes	Yes	Yes	Yes	Yes	Unsure	Yes	Yes	Yes	Yes	**9/10**
Maddigan, 2006	Yes	Yes	Yes	Unsure	Unsure	Yes	Yes	Yes	Yes	Yes	**8/10**
Wexler, 2006	Yes	Yes	Yes	Yes	Yes	Yes	Yes	Yes	Yes	Yes	**10/10**
Clarke, 2006	Yes	Yes	Yes	Yes	Yes	Yes	Yes	Yes	Yes	Yes	**10/10**
Smith, 2008	Yes	Yes	Unsure	Yes	Yes	Yes	Yes	Yes	Yes	Yes	**9/10**
Lloyd, 2008	Yes	Yes	Unsure	Yes	Unsure	Yes	Yes	Yes	Yes	Yes	**8/10**
Marrett, 2011	Yes	Yes	Yes	Yes	Unsure	Yes	Yes	Unsure	Yes	Yes	**8/10**
Quah, 2011	Yes	Yes	Yes	Yes	Yes	Yes	Yes	Unsure	Yes	Yes	**9/10**
O'Reilly, 2011	Yes	Yes	No	No	Yes	No	Yes	Yes	Yes	Yes	**7/10**
Lee, 2012	Yes	Yes	Yes	Yes	Yes	Yes	Yes	Yes	Yes	Yes	**10/10**
Zhang, 2012	Yes	Yes	Yes	Unsure	Yes	Yes	Yes	Yes	Yes	Yes	**9/10**
Luk, 2014	Yes	Yes	Yes	Yes	Unsure	No	Yes	Yes	Yes	Yes	**8/10**
Harris, 2014	Yes	Yes	Yes	Yes	Unsure	Yes	Yes	Yes	Yes	Yes	**9/10**
Kiadaliri, 2014	Yes	Yes	Yes	Yes	Yes	Yes	Yes	Yes	Yes	Yes	**10/10**
Pan, 2015	Yes	Yes	Unsure	Yes	Yes	Yes	Yes	Yes	Yes	Yes	**9/10**
Hayes, 2016	Yes	Yes	Yes	Yes	Yes	Yes	Yes	Yes	Yes	Yes	**10/10**
Jiao, 2017	Yes	Yes	Yes	Yes	Yes	Yes	Yes	Yes	Yes	Yes	**10/10**
Riandini, 2018	Yes	Yes	Yes	Yes	Yes	No	Yes	Yes	Yes	Yes	**9/10**
Pan, 2018	Yes	Yes	Unsure	Yes	Yes	Yes	Yes	Yes	Yes	Yes	**9/10**
Takahara, 2019	Yes	Yes	Unsure	Unsure	Yes	Yes	Yes	Yes	Yes	Yes	**8/10**
Shao, 2019	Yes	Yes	Yes	Yes	Yes	Yes	Yes	Yes	Yes	Yes	**10/10**
Pham, 2020	Yes	Yes	Yes	Yes	Yes	Yes	Yes	Yes	Yes	Yes	**10/10**
Yfantopoulos, 2019	Yes	Yes	Yes	Yes	Yes	No	Yes	Yes	Yes	Yes	**9/10**
Zhang, 2020	Yes	Yes	Yes	Unsure	Yes	Yes	Yes	Yes	Yes	Yes	**9/10**
Keng, 2021	Yes	Yes	Yes	Yes	Yes	Yes	Yes	Yes	Yes	Yes	**10/10**
Chen, 2021	Yes	Yes	Yes	Yes	Yes	No	Yes	Yes	Yes	Yes	**9/10**
Chao, 2021	Yes	Yes	Yes	Unsure	Unsure	Yes	Yes	Yes	Yes	Yes	**8/10**
Neuwahl, 2021	Yes	Yes	Yes	Yes	Yes	Yes	Yes	Yes	Yes	Yes	**10/10**
Laxy, 2021	Yes	Yes	Yes	Unsure	Yes	Yes	Yes	Yes	Yes	Yes	**9/10**
Kuo, 2021	Yes	Yes	Unsure	Yes	No	Yes	Yes	Yes	Yes	Yes	**8/10**
**Total**	**35/35**	**35/35**	**28/35**	**27/35**	**27/35**	**27/35**	**35/35**	**33/35**	**35/35**	**35/35**	**--**
