# Supplementary material for: Health state utility values ranges across varying stages and severity of type 2 diabetes-related complications: A systematic review
Source: PLoS One. 2024 Apr 4;19(4):e0297589. doi: 10.1371/journal.pone.0297589 (PMC10994347; doi:10.1371/journal.pone.0297589)
Supplement: S4 Table — (PDF) [file pone.0297589.s005.pdf]

S4 Table : HSUV decrement and definition for cardiovascular complication

Author (Year)	Angina (95% CI)	Myocardial Infarction (95% CI)	Not defined (95% CI)	Definition by authors
Clarke (2002)	-0.090 (-0.126, -0.054)	-0.055 (-0.067, -0.042)	-	-
Neuwahl (2021)	-0.042 (NR)	-0.018 (NR)	-	-
Takahara (2019)	-0.031 (SE 0.012)	-	-	Coronary artery disease with cardiac symptoms
Lee (2012)	-0.027 (SE 0.0114)	-0.007 (SE 0.0148)	-	-
Kiadaliri (2014)	-0.025 (NR)	-0.012 (NR)	-	-
Chao Yun Li (2020)	-0.017 (-0.028, -0.006)	-0.005 (-0.18, 0.008)	-	-
Hayes (2016)	-0.010 (-0.025, 0.005)	-0.026 (-0.047, -0.004)	-	-
O'reilly (2011)	-	-0.059 (0.0168)	-	-
Yfantoupoulos (2019)	-	-0.038 (-0.071, -0.002)	-	-
Ping Zhang (2012)	-	-0.019 (SE 0.005)	-	Percutaneous coronary intervention, angiogram, treatment
Shao (2019)	-	-0.018 (-0.054,0.017)	-	-
Kuo (2021)	-	-	-0.1850 (SE 0.076)	other coronary heart disease
Maddigan (2006)	-	-	-0.0800 (-0.11, -0.05)	heart disease
Pan (2016)	-	-	-0.0740 (-0.122-0.018)	heart disease
Quah (2011)	-	-	-0.0500 (NR)	ischemic heart disease
Pham (2020)	-	-	-0.0500 (-0.14, 0.04)	diabetic heart disease
Chen (2021)	-	-	-0.0500 (-0.12, 0.02)	IHD
Solli (2010)	-	-	-0.0370 (-0.103, 0.03)	IHD (MI and angina)
Luk (2014)	-	-	-0.0340 (SE 0.004)	coronary heart disease
Bagust (2005)	-	-	-0.0280 (SE 0.01)	coronary heart disease (MI, angina, heart failure)
Zhang Yi (2020)	-	-	-0.0280 (SE 0.005)	coronary heart disease
Tabaei (2004)	-	-	-0.0240 (SE 0.011)	heart disease
Jiao (2017)	-	-	-0.0170 (-0.042, 0.08)	heart disease
Wexler (2006)	-	-	-0.0100 (SE 0.02)	coronary heart disease
Pan (2018)	-	-	-0.0080 (-0.016, -0.001)	heart disease
