# Supplementary material for: Health state utility values ranges across varying stages and severity of type 2 diabetes-related complications: A systematic review
Source: PLoS One. 2024 Apr 4;19(4):e0297589. doi: 10.1371/journal.pone.0297589 (PMC10994347; doi:10.1371/journal.pone.0297589)
Supplement: S5 Table — (PDF) [file pone.0297589.s006.pdf]

S5 Table : HSUV decrement and definition for stroke complication

Author (Year)	Mild stroke (95% CI)	Severe (with sequelae) (95% CI)	Undefined (95% CI)	Definition by authors
Kuo (2021)	-0.078 (SE 0.022)	-0.266 (SE 0.041)	-	Severe stroke=residual deficit
Lee (2012)	-0.0684 (SE 0.0242)	-0.0761 (SE 0.0133)	-	TIA; ischemic stroke
Keng (2022)	-0.057 (-0.086,0.028)	-0.164 (-0.251, -0.076)	-	TIA, intracranial bleed
Coffey (2002)	-0.044 (SE 0.012)	-0.072 (SE 0.016)	-	TIA; stroke with residual
Zhang (2012)	-0.029 (SE 0.015)	-0.035 (SE 0.01)	-	TIA; cerebrovascular accident
Takahara (2019)	-0.006 (SE 0.009)	-0.098 (SE 0.018)	-	sequelae free; with sequelae
Chen (2021)	-	-0.211 (-0.32, -0.10)	-	chronic stroke
Clarke (2002)	-	-0.164 (-0.222, -0.105)	-	stroke
Maddigan (2006)	-	-0.150 (-0.21, -0.10)	-	stroke with sequelae
Shao (2019)	-	-	-0.2020 (NR)	stroke
Pan (2016)	-	-	-0.1600 (-0.287, -0.030)	cerebrovascular accident
Laxy (2021)	-	-	-0.1220 (SE 0.018)	stroke
Bagust (2005)	-	-	-0.1150 (SE 0.017)	stroke and TIA
Zhang Yi (2020)	-	-	-0.1010 (SE 0.006)	stroke
Hayes (2016)	-	-	-0.0990 (-0.117, -0.081)	stroke
Tabaei (2004)	-	-	-0.0870 (SE 0.028)	stroke
Yfantoupoulos (2019)	-	-	-0.0820 (-0.170, -0.003)	stroke
Quah (2011)	-	-	-0.0700 (NR)	stroke
Kiadaliri (2014)	-	-	-0.0590 (NR)	stroke
Chao Yun Li (2020)	-	-	-0.0470 (-0.057, -0.038)	stroke
O'reilly (2011)	-	-	-0.0462 (SE 0.0233)	stroke
Neuwahl (2021)	-	-	-0.0440 (NR)	stroke
Jiao (2017)	-	-	-0.0420 (-0.072, -0.012)	stroke
Wexler (2006)	-	-	-0.0200 (SE 0.02)	stroke
