# Supplementary material for: Health state utility values ranges across varying stages and severity of type 2 diabetes-related complications: A systematic review
Source: PLoS One. 2024 Apr 4;19(4):e0297589. doi: 10.1371/journal.pone.0297589 (PMC10994347; doi:10.1371/journal.pone.0297589)
Supplement: S6 Table — (PDF) [file pone.0297589.s007.pdf]

S6 Table : HSUV decrement and definition for nephropathy complication

Author/Year	Proteinurea (95% CI)	Broadly defined Nephropathy (95% CI)	ESRF/Dialysis (95% CI)	Definition by authors
Bagust (2005)	-0.048 (SE 0.022)	-	-0.175 (SE 0.028)	Proteinurea; ESRF
Kuo (2021)	-	-	-0.148 (SE 0.05)	Dialysis; transplant
Chen (2021)	-	-	-0.135 (SE 0.038)	ESRF
Neuwahl (2021)	-	-0.0190 (NR)	-0.105 (NR)	eGFR<60mL/min; dialysis
Coffey (2002)	-	-0.011 (SE 0.009)	-0.078 (SE 0.026)	Diabetic kidney disease; dialysis
Ping Zhang (2012)	-	-	-0.060 (SE 0.027)	dialysis
Jiao (2017)	-	-0.011 (-0.029, 0.006)	-0.055 (-0.093, -0.017)	nephropathy, ESRF
Takahara (2019)	-0.017 (SE 0.007)	-	-0.050 (SE 0.021)	overt nephro, dialysis
O'reilly (2011)	-	-0.1018 (SE 0.0472)	-	kidney failure
Pham (2020)	-	-0.080 (-0.23, 0.07)	-	nephropathy
Keng (2022)	-0.018 (-0.031, -0.005)	-0.072 (-0.089, -0.054)	-0.08 (-0.108, -0.051)	Albuminuria; >45<60mL/min; ESRF
Zhang Yi (2020)	-	-0.058 (SE 0.006)	-	nephropathy
Hayes (2016)	-	-0.049 (-0.084, -0.015)	-	renal failure
Kiadaliri (2014)	-	-0.0406 (NR)	-	kidney disorders
Laxy (2021)	-	-0.032 (SE 0.025)	-	nephropathy
Shao (2019)	-	-0.029 (NR)	-	Creatinine >291 umol/L, dialysis, transplant
Yfantoupoulos (2019)	-	-0.018 (-0.072, 0.04)	-	diab nephropathy
Luk (2014)	-0.014 (0.003)		-	nephropathy = albuminuria/CKD
Lee (2012)	-	-0.0044 (SE 0.0167)	-	nephropathy
Pan (2018)	-	-0.003 (-0.013, 0.008)	-	nephropathy
