# Supplementary material for: Health state utility values ranges across varying stages and severity of type 2 diabetes-related complications: A systematic review
Source: PLoS One. 2024 Apr 4;19(4):e0297589. doi: 10.1371/journal.pone.0297589 (PMC10994347; doi:10.1371/journal.pone.0297589)
Supplement: S7 Table — (PDF) [file pone.0297589.s008.pdf]

S7 Table : HSUV decrement and definition for retinopathy complication

Author/Year	Mild (95% CI)	Moderate (95% CI)	Severe (blind) (95% CI)	Undefined (95% CI)	Definition by authors
Lloyd (2008)	-0.086 (SE 0.01) Mild visual loss 6/12-6/18	-0.1845 (SE 0.014) Visual loss 6/60-6/120	-0.2434 (SE 0.015) Counting fingers/hand motion	-	reference: Worst Seeing Eye (WSE)
Tung (2005)	-0.0630 (-0.096, -0.031)	-0.1040 (-0.170, -0.039)	-0.1130 (-0.188, -0.039)	-	NPDR, PDR, blind
Philip Clarke (2006)	-0.0050 (SE 0.009) LogMAR > 0 WSE	-0.0070 (SE 0.062) LogMAR ≤ 0 WSE	-0.0540 (SE 0.018) LogMAR > 0 BSE (legally blind)	-	LogMAR score ≤ 0.0 = normal / better than normal corrected vision
Smith (2008)	-0.0300 (-0.05, -0.01) Visual acuity = 20/40	-0.0600 (-0.10, -0.02) Visual acuity ≤ 20/80	-	-	doubling visual acuity based on BSE
Pan (2018)	-0.0130 (-0.029, 0.005)	-0.0190 (-0.037, -0.002)	-	-	Unilateral, bilateral DR
Coffey (2002)	-	-	-0.1700 (SE 0.011)	-	bilateral blind
Takahara (2019)	-	-0.0230 (SE 0.011)	-0.0950 (SE 0.026)	-	PDR, bilateral blind
Chen (2021)	-	-	-0.2030 (-0.37,-0.04)	-	Diabetic retinopathy
Tabaei (2004)	-	-	-0.1090 (SE 0.012)	-	blind
Laxy (2021)	-	-	-0.0940 (SE 0.056)	-	blind
Hayes (2016)	-	-	-0.0830 (-0.131, -0.034)	-	Blindness in Worse eye
Clarke (2002)	-	-	-0.0740 (-0.124, -0.052)	-	unilateral blind
Bagust (2005)	-	-	-0.0570 (SE 0.022)	-	proliferative retinopathy
Jiao (2017)	-	-	-0.0430 (-0.075, 0.010)	-	STDR (sight threatening)
Shao (2019)	-	-	-0.0390 (NR)	-	severe vision loss
Chao (2020)	-	-	-0.0230 (-0.030, 0.017)	-	blindness
Neuwahl (2021)	-	-	-0.0140 (NR)	-	retinal laser photocoagulation
Pham (2020)	-	-	-	-0.1700 (-0.37, 0.03)	retinopathy
Yfantoupoulos (2019)	-	-	-	-0.0660 (-0.139, -0.005)	retinopathy
Kiadaliri (2014)	-	-	-	-0.0103 (NR)	retinopathy
Quah (2011)	-	-	-	-0.0400 (NR)	eye disease
Zhang Yi (2020)	-	-	-	-0.0220 (SE 0.005)	retinopathy
Lee (2012)	-	-	-	-0.0217 (SE 0.0093)	retinopathy
Pan (2016)	-	-	-	-0.0160 (-0.056, 0.027)	retinopathy
