# Supplementary material for: Health state utility values ranges across varying stages and severity of type 2 diabetes-related complications: A systematic review
Source: PLoS One. 2024 Apr 4;19(4):e0297589. doi: 10.1371/journal.pone.0297589 (PMC10994347; doi:10.1371/journal.pone.0297589)
Supplement: S8 Table — (PDF) [file pone.0297589.s009.pdf]

S8 Table : HSUV decrement and definition for neuropathy complication

Author/Year	Severe neuropathy (95% CI)	Neuropathy undefined (95% CI)	Definition by authors
Solli (2010)	-0.187 (-0.316, -0.057)	-	neuropathy (with pain)
Ping Zhang (2012)	-0.105 (SE 0.005)	-0.0390 (SE 0.005)	not pain/painful neuro
Riandini (2019)	-0.1000 (-0.15, -0.006)	-	burning neuropathy
Takahara (2019)	-0.0700 (SE 0.001)	-0.0440 (SE 0.006)	symptomatic neuro; claudication (pain)
Coffey (2002)	-0.06 (SE 0.01)	-0.0650 (SE 0.008)	neuropathy; symptomatic tingling/burning
Bagust (2005)	-	-0.0840 (SE 0.014)	neuropathy
Tabaei (2004)	-	-0.0720 (SE 0.01)	neuro
Laxy (2021)	-	-0.0670 (SE 0.02)	neuropathy
Luk (2014)	-	-0.0630 (SE 0.007)	peripheral sensory neuro
Kuo (2021)	-	-0.0620 (SE 0.018)	diab neuropathy
Pan (2016)	-	-0.0570 (-0.093. -0.017)	neuropathy
Yfantoupoulos (2019),	-	-0.0510 (-0.121, 0.02)	diab neuropathy
Quah (2011)	-	-0.0500 (NR)	peripheral neuropathy
Shao (2019)	-	-0.0240 (-0.080, -0.053)	severe pressure loss (no pain)
Chao Yun Li (2020)	-	-0.0140 (-0.017, -0.010)	neuropathy
