# Supplementary material for: Health state utility values ranges across varying stages and severity of type 2 diabetes-related complications: A systematic review
Source: PLoS One. 2024 Apr 4;19(4):e0297589. doi: 10.1371/journal.pone.0297589 (PMC10994347; doi:10.1371/journal.pone.0297589)
Supplement: S9 Table — (PDF) [file pone.0297589.s010.pdf]

S9 Table: HSUV decrement and definition for foot ulcer and amputation

Author / Year	Foot Ulcer (95% CI)	Amputation (95% CI)	Definition by authors
Bagust (2005)	-0.1700 (SE 0.019)	-0.2720 (SE 0.029)	foot ulcer; amputation
Chen (2021)	-0.1360 (SE 0.05)	-0.2760 (SE 0.169)	diabetic foot disease
Takahara (2019)	-0.1400 (SE 0.034)	-0.1770 (SE 0.058)	foot ulcer, gangrene; amputation
Tabaei (2004)	-0.1120 (SE 0.014)	-0.1140 (SE 0.034)	peripheral vasc disease; amputation
Coffey (2002)	-0.0990 (SE 0.013)	-0.1050 (SE 0.022)	sores; amputation
Zhang Yi (2020)	-0.1180 (SE 0.009)	-	diabetic foot
Quah (2011)	-0.0800 (NR)	-	peripheral vascular disease
Kuo (2021)	-	-0.2880 (SE 0.079)	amputation
Clarke (2002)	-	-0.2800 (-0.389, -0.170)	amputation
Keng (2022)	-	-0.2060 (-0.259, -0.152)	amputation (lower limb)
Neuwahl (2021)	-	-01630 (NR)	amputation
Chao Yun Li (2020)	-	-0.1360 (-0.154, -0.119)	diab foot + amputation
Hayes (2016)	-	-0.1220 (-0.175, -0.069)	amputation of toe or limb
Ping Zhang (2012)	-	-0.1080 (SE 0.017)	amputation
O'reilly (2011)	-	-0.0631 (SE 0.0586)	amputation
