# Supplementary material for: Health state utility values ranges across varying stages and severity of type 2 diabetes-related complications: A systematic review
Source: PLoS One. 2024 Apr 4;19(4):e0297589. doi: 10.1371/journal.pone.0297589 (PMC10994347; doi:10.1371/journal.pone.0297589)
Supplement: S10 Table — (PDF) [file pone.0297589.s011.pdf]

S10 Table: HSUV decrement and definition for hypoglycemia complication

	Mild (95% CI)	Moderate (95% CI)	Severe (95% CI)	Very Severe (95% CI)
Harris (2014)	-0.0028 (0.0001, -0.0057)<br>non severe day time	-0.0076 (-0.0046, -0.0109)<br>non severe nocturnal	-0.0726 (0.0493, -0.0983)<br>severe daytime event	-0.0826 (-0.0568, -0.1103)<br>severe nocturnal event
Tabaei (2004)	-0.0190 (NR)<br>1-4 x hypo	-0.0380 (NR)<br>5-8 x hypo	-0.057 (NR)<br>9-12 x hypo	-0.076 (NR)<br>12+ hypo
Marrett (2011)	-0.0100 (NR)<br>mild (no interruption of activities)	-0.0600 (NR)<br>moderate (some interruption)	-0.13 (NR)<br>severe (need assistance of others)	-0.21 (NR)<br>very severe<br>(need medical attention)
Yfantoupoulos (2019)	-	-	-	-0.05 (-0.091, -0.009)<br>severe hypo (at least one event needing hosp)
Shao (2019)	-	-	-	-0.0360 (-0.056, -0.016)<br>symptomatic and severe
Takahara (2019)	-	-	-	-0.0250 (SE 0.012)<br>severe/nocturnal hypo
Zhang Yi (2020)	-0.0070 (SE 0.002)<br>Symptomatic hypoglycemia	-		-0.008 (SE 0.004)<br>severe hypoglycemia
Luk (2014)	-	-	-0.0400 (SE 0.005)<br>hypoglycemia > 1 x monthly	-
Chen (2021)	-0.0090 (SE 0.018)<br>Symptomatic, acute event<br>(previous month only)	-		-
Neuwahl (2021)	-	-	-0.0060 (NR)<br>hypoglycemia<br>(any assistance)	-
