# Supplementary material for: Health state utility values ranges across varying stages and severity of type 2 diabetes-related complications: A systematic review
Source: PLoS One. 2024 Apr 4;19(4):e0297589. doi: 10.1371/journal.pone.0297589 (PMC10994347; doi:10.1371/journal.pone.0297589)
Supplement: S1 File — (PDF) [file pone.0297589.s012.pdf]

Abbreviations :

CHD – coronary heart disease

CI – confidence interval

CKD – chronic kidney disease

COPD- Chronic obstructive pulmonary disease

DR – diabetic retinopathy

ED-5D-5L - 5-level EQ-5D version

eGFR – estimated glomerular filtration rate

ESRF/ESRD – end stage renal failure/end stage renal disease

EU- European Union

HbA1c – Glycated hemoglobin

HRQoL – health related quality of life

HSUV – health state utility value

HUI-3 - Health Utilities Index Mark

IHD – ischemic heart disease

MI – myocardial infarction

NR – Not reported

NR- Not reported

OHA - Oral hypoglycemic agents

OLS- Ordinary Least Squares

PAD – peripheral artery disease

PDR – proliferative diabetic retinopathy

QWB-SA - Self-administered Quality of Well-Being Index

SE – standard error

SF-6D - Short-form 6-dimensional

SG - standard gamble

T2DM-Type 2 diabetes mellitus

TIA – transient ischemic attack

TTO - time-trade off

U.S- United States
