# Supplementary material for: Health state utility values ranges across varying stages and severity of type 2 diabetes-related complications: A systematic review
Source: PLoS One. 2024 Apr 4;19(4):e0297589. doi: 10.1371/journal.pone.0297589 (PMC10994347; doi:10.1371/journal.pone.0297589)
Supplement: S2 File — (PDF) [file pone.0297589.s013.pdf]

S2: Data extraction form items

Study background

1. Study Reference ID
2. Journal name
3. Year of publication
4. Title of study
5. Authorship

Study characteristics

1. Country of study
2. Tariff used
3. Ethnicity
4. Aim of study
5. Study design
6. Start date/End date
7. Possible conflicts of interest for study authors

Sampling (population/ patient characteristics)

1. Population description
2. Inclusion criteria
3. Exclusion criteria
4. Age
5. HbA1c (mean, standard deviation)
6. Comorbidities

Data source/collection (other issues)

1. Missing data (how is it dealt with)
2. Incomplete outcome or other potential problems
3. Respondent selection and recruitment
4. Response rate
5. Sample size

General population norms

1. Baseline population characteristics
2. Participant characteristics where HRQoL were captured
3. Methods of valuation: participant characteristics used in valuation of HRQoL change
4. Setting (what type of outpatient setting/general survey)

Other details

1. Timing of data collection
2. Treatment type (oral or injection)

Determination of HSUV/descriptive system

1. Details of health state description system
2. Valuation technique

Health states valued (CREATE guideline)

1. Staging or severity of complications
2. Self-reported complications or documented from medical records
3. Macrovascular complication (cardiovascular complication, heart failure, stroke)
4. Microvascular complication (nephropathy, retinopathy, foot ulcer, neuropathy) and other relevant complications (hypoglycemia)
5. Synthesis of HSUV in study
6. Baseline HSUV in general population with dm without complication
7. Mean and variance around HSUV used in model
8. Reasons for excluding observations /analysis

Modeling

1. Dependent variable for model – adjustment done?
2. Model specs (choosing variables)
3. Model estimator
4. Statistical method used
5. HSUV summary

Adapted from: Identification, Review and Use of Health State Utilities in Cost-effectiveness Models : An ISPOR Good Practices for Outcomes Research Task Force Report. John Brazier
