# Supplementary material for: Health state utility values ranges across varying stages and severity of type 2 diabetes-related complications: A systematic review
Source: PLoS One. 2024 Apr 4;19(4):e0297589. doi: 10.1371/journal.pone.0297589 (PMC10994347; doi:10.1371/journal.pone.0297589)
Supplement: S3 File — (PDF) [file pone.0297589.s014.pdf]

S3: Risk of bias assessment criteria

1.	Study Purpose	:	Was the purpose stated clearly
2.	Justification of study	:	Was relevant background literature reviewed?
3.	Design	:	Randomized Control Trial (RCT) /Cohort/cross-sectional
4.	Sample size/ Respondent selection	:	Was sample size described in detail-how was sampling done, how many, who, where? justified-ethic procedure done, informed consent? Is the study representative to the population from which they were recruited? if more than 1 group, similarity exist between groups?
5.	Inclusion/exclusion criteria	:	Does the study make clear the inclusion/exclusion criteria?
6.	Response rates to instrument used	:	Are response rates reported. If reported, rates unlikely to be a threat to validity? Drop outs reported/missing data? drop out size is small? Will the size of loss to follow up NOT likely affect validity of estimates? Loss to follow-up
7.	Appropriateness Tariff	:	Is the tariff reported and does it match study population
8.	Outcome measures	:	Is the measure used valid in the group of patients? were the objective, standard criteria used for measurement of the condition? if there were multiple outcomes, was that considered for statistical analysis?
9.	Uncertainty measurement		Is there any reported uncertainty measurement (e.g., 95% confidence interval, standard error)? Were confounding factors identified? Strategies to deal with confounding factors?
10.	Outcome measures		Were outcome measures reliable and valid?
11.	Results		Results significant? Analysis method appropriate? What was clinical importance of results?
12.	Conclusions and Implications		Conclusions were appropriate given study methods and results. what did study conclude? implications for practice? limitation or bias in study?

Adapted from : Papaioannou, Chiu, NICE, McMaster Uni
