# Supplementary material for: Health state utility values ranges across varying stages and severity of type 2 diabetes-related complications: A systematic review
Source: PLoS One. 2024 Apr 4;19(4):e0297589. doi: 10.1371/journal.pone.0297589 (PMC10994347; doi:10.1371/journal.pone.0297589)
Supplement: S4 File — (PDF) [file pone.0297589.s015.pdf]

S3(A) Table: Risk of bias Table

Study ID	Purpose	Justification	Sample size	Inclusion/ exclusion	Response rates	Appropriate Tariff	Outcome measures	Uncertainty measurement	Results	Conclusions/ Implications	Total points
Clarke, 2002	Yes	Yes	Yes	Yes	Yes	Yes	Yes	Yes	Yes	Yes	**10/10**
Coffey, 2002	Yes	Yes	Yes	Yes	Yes	Unsure	Yes	Yes	Yes	Yes	**9/10**
Tabaei, 2004	Yes	Yes	Yes	No	No	Unsure	Yes	Yes	Yes	Yes	**7/10**
Bagust, 2005	Yes	Yes	Yes	Unsure	Unsure	Yes	Yes	Yes	Yes	Yes	**8/10**
Tung, 2005	Yes	Yes	Yes	Yes	Yes	Unsure	Yes	Yes	Yes	Yes	**9/10**
Maddigan, 2006	Yes	Yes	Yes	Unsure	Unsure	Yes	Yes	Yes	Yes	Yes	**8/10**
Wexler, 2006	Yes	Yes	Yes	Yes	Yes	Yes	Yes	Yes	Yes	Yes	**10/10**
Clarke, 2006	Yes	Yes	Yes	Yes	Yes	Yes	Yes	Yes	Yes	Yes	**10/10**
Smith, 2008	Yes	Yes	Unsure	Yes	Yes	Yes	Yes	Yes	Yes	Yes	**9/10**
Lloyd, 2008	Yes	Yes	Unsure	Yes	Unsure	Yes	Yes	Yes	Yes	Yes	**8/10**
Marrett, 2011	Yes	Yes	Yes	Yes	Unsure	Yes	Yes	Unsure	Yes	Yes	**8/10**
Quah, 2011	Yes	Yes	Yes	Yes	Yes	Yes	Yes	Unsure	Yes	Yes	**9/10**
O'Reilly, 2011	Yes	Yes	No	No	Yes	No	Yes	Yes	Yes	Yes	**7/10**
Lee, 2012	Yes	Yes	Yes	Yes	Yes	Yes	Yes	Yes	Yes	Yes	**10/10**
Zhang, 2012	Yes	Yes	Yes	Unsure	Yes	Yes	Yes	Yes	Yes	Yes	**9/10**
Luk, 2014	Yes	Yes	Yes	Yes	Unsure	No	Yes	Yes	Yes	Yes	**8/10**
Harris, 2014	Yes	Yes	Yes	Yes	Unsure	Yes	Yes	Yes	Yes	Yes	**9/10**
Kiadaliri, 2014	Yes	Yes	Yes	Yes	Yes	Yes	Yes	Yes	Yes	Yes	**10/10**
Pan, 2015	Yes	Yes	Unsure	Yes	Yes	Yes	Yes	Yes	Yes	Yes	**9/10**
Hayes, 2016	Yes	Yes	Yes	Yes	Yes	Yes	Yes	Yes	Yes	Yes	**10/10**
Jiao, 2017	Yes	Yes	Yes	Yes	Yes	Yes	Yes	Yes	Yes	Yes	**10/10**
Riandini, 2018	Yes	Yes	Yes	Yes	Yes	No	Yes	Yes	Yes	Yes	**9/10**
Pan, 2018	Yes	Yes	Unsure	Yes	Yes	Yes	Yes	Yes	Yes	Yes	**9/10**
Takahara, 2019	Yes	Yes	Unsure	Unsure	Yes	Yes	Yes	Yes	Yes	Yes	**8/10**
Shao, 2019	Yes	Yes	Yes	Yes	Yes	Yes	Yes	Yes	Yes	Yes	**10/10**
Pham, 2020	Yes	Yes	Yes	Yes	Yes	Yes	Yes	Yes	Yes	Yes	**10/10**
Yfantopoulos, 2019	Yes	Yes	Yes	Yes	Yes	No	Yes	Yes	Yes	Yes	**9/10**
Zhang, 2020	Yes	Yes	Yes	Unsure	Yes	Yes	Yes	Yes	Yes	Yes	**9/10**
Keng, 2021	Yes	Yes	Yes	Yes	Yes	Yes	Yes	Yes	Yes	Yes	**10/10**
Chen, 2021	Yes	Yes	Yes	Yes	Yes	No	Yes	Yes	Yes	Yes	**9/10**
Chao, 2021	Yes	Yes	Yes	Unsure	Unsure	Yes	Yes	Yes	Yes	Yes	**8/10**
Neuwahl, 2021	Yes	Yes	Yes	Yes	Yes	Yes	Yes	Yes	Yes	Yes	**10/10**
Laxy, 2021	Yes	Yes	Yes	Unsure	Yes	Yes	Yes	Yes	Yes	Yes	**9/10**
Kuo, 2021	Yes	Yes	Unsure	Yes	No	Yes	Yes	Yes	Yes	Yes	**8/10**
**Total**	**35/35**	**35/35**	**28/35**	**27/35**	**27/35**	**27/35**	**35/35**	**33/35**	**35/35**	**35/35**	**--**
