# Supplementary material for: Health state utility values ranges across varying stages and severity of type 2 diabetes-related complications: A systematic review
Source: PLoS One. 2024 Apr 4;19(4):e0297589. doi: 10.1371/journal.pone.0297589 (PMC10994347; doi:10.1371/journal.pone.0297589)
Supplement: S1 Fig — (PDF) [file pone.0297589.s016.pdf]

S ***1 Figure: Overview of HSUV decrement ranges by reporting methods (Self-report or from medical reports)***

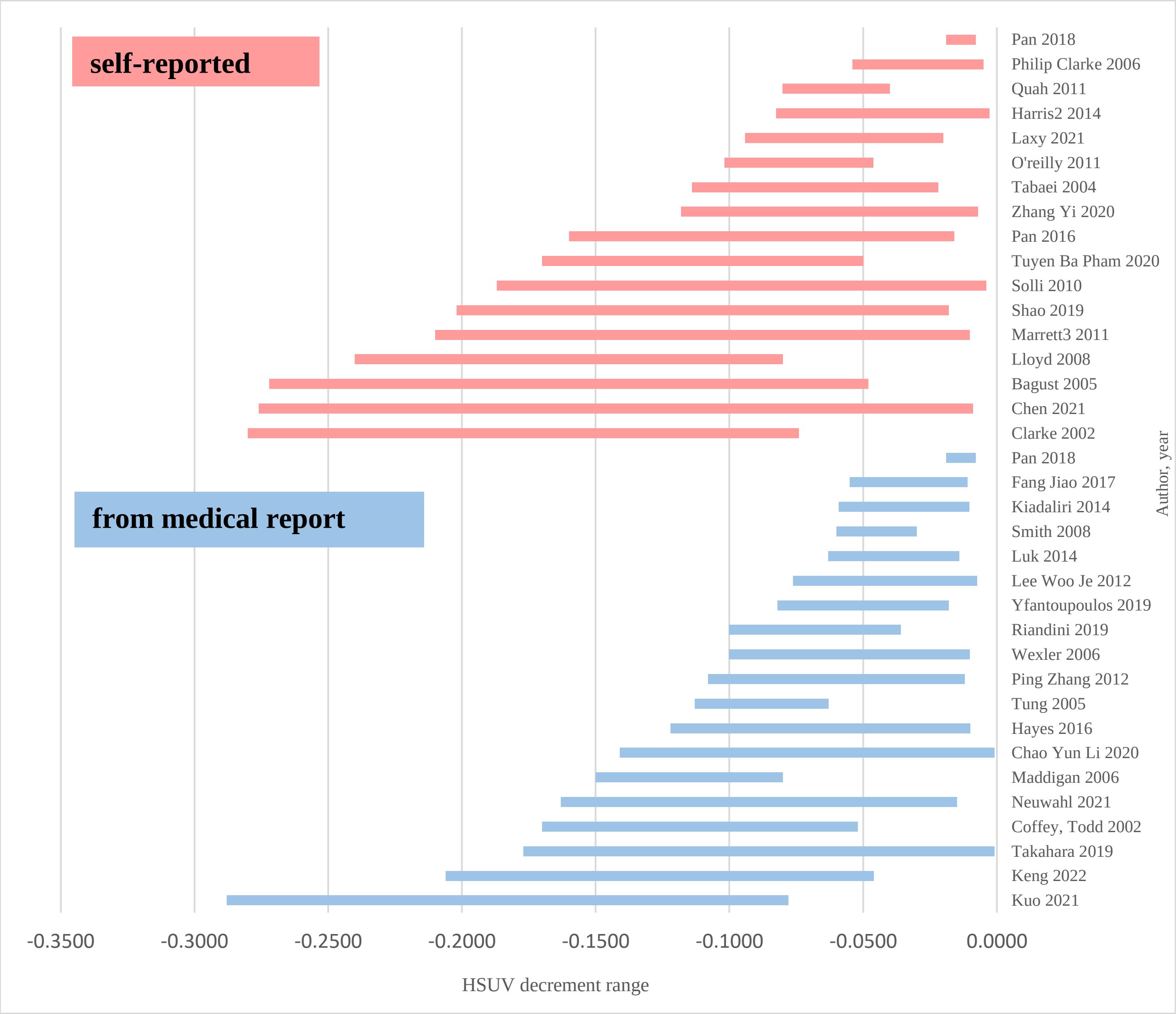

Note: Larger and wider HSUV decrement ranges were seen when complication details were reported by patients who were experiencing the complications themselves.
