# Supplementary material for: Health state utility values ranges across varying stages and severity of type 2 diabetes-related complications: A systematic review
Source: PLoS One. 2024 Apr 4;19(4):e0297589. doi: 10.1371/journal.pone.0297589 (PMC10994347; doi:10.1371/journal.pone.0297589)
Supplement: S2 Fig — (PDF) [file pone.0297589.s017.pdf]

S2 Figure. HSUV decrement for heart failure complication

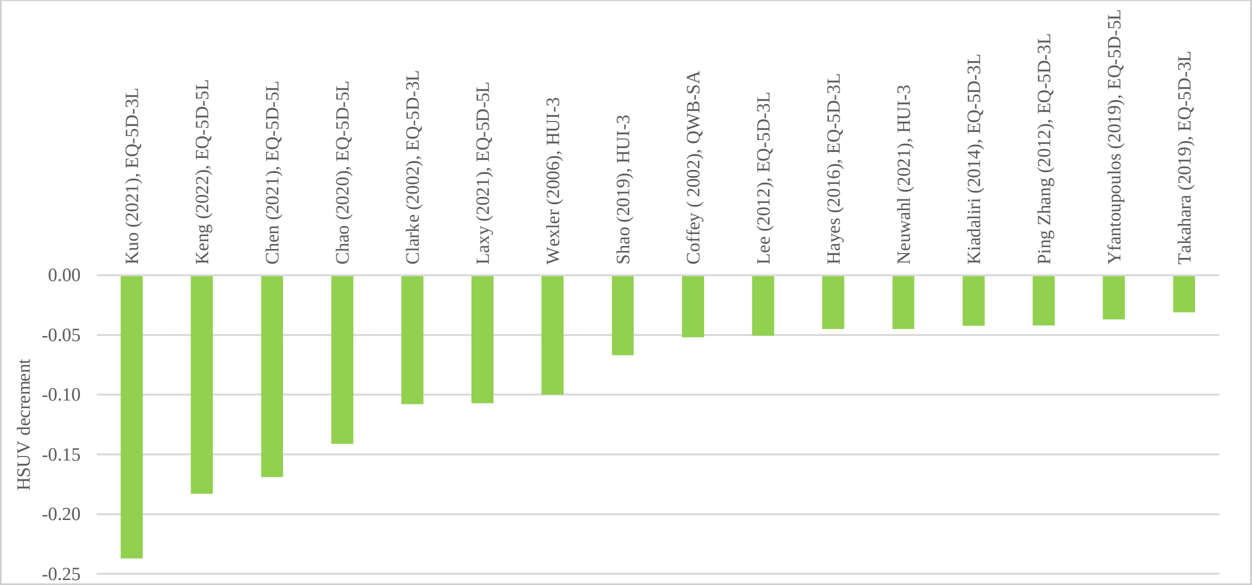

**Author (year)**	**Heart failure (95% CI)**
Kuo (2021)	-0.237 (SE 0.03)
Keng (2022)	-0.183 (-0.258, -0.108)
Chen (2021)	-0.169 (-0.25, -0.09)
Chao Yun Li (2020)	-0.141 (-0.161, -0.121)
Clarke (2002)	-0.108 (-0.169, -0.048)
Laxy (2021)	-0.107 (SE 0.019)
Wexler (2006)	-0.1 (SE 0.03)
Shao (2019)	-0.067 (NR)
Coffey (2002)	-0.052 (SE 0.011)
Lee (2012)	-0.0505 (SE 0.0154)
Hayes (2016)	-0.045 (-0.066, -0.025)
Neuwahl (2021)	-0.045 (NR)
Kiadaliri (2014)	-0.0422 (NR)
Ping Zhang (2012)	-0.042 (SE 0.008)
Yfantoupoulos (2019)	-0.037 (-0.171, 0.078)
Takahara (2019)	-0.031 (SE 0.012)
